# Efficacy of MyPEEPS Mobile, an HIV Prevention Intervention Using Mobile Technology, on Reducing Sexual Risk Among Same-Sex Attracted Adolescent Males: A Randomized Clinical Trial

**DOI:** 10.1001/jamanetworkopen.2022.31853

**Published:** 2022-09-21

**Authors:** Rebecca Schnall, Lisa M. Kuhns, Cynthia Pearson, D. Scott Batey, Josh Bruce, Marco A. Hidalgo, Sabina Hirshfield, Patrick Janulis, Haomiao Jia, Asa Radix, Uri Belkind, Rafael Garibay Rodriguez, Robert Garofalo

**Affiliations:** 1 School of Nursing, Columbia University, New York, New York; 2 Heilbrunn Department of Population and Family Health, Mailman School of Public Health, Columbia University, New York, New York; 3 Division of Adolescent and Young Adult Medicine, Ann & Robert H. Lurie Children’s Hospital of Chicago, Chicago, Illinois; 4 Department of Pediatrics, Northwestern University, Feinberg School of Medicine, Chicago, Illinois; 5 Indigenous Wellness Research Institute, School of Social Work, University of Washington, Seattle; 6 Department of Social Work, University of Alabama at Birmingham; 7 Birmingham AIDS Outreach, Birmingham, Alabama; 8 Division of General Internal Medicine and Health Services Research, Medicine–Pediatrics Section, Department of Medicine, David Geffen School of Medicine at UCLA, Los Angeles, California; 9 STAR Program, Department of Medicine, SUNY Downstate Health Sciences University, Brooklyn, New York; 10 Department of Medical Social Sciences, Northwestern University, Chicago, Illinois; 11 Callen-Lorde Community Health Center, New York, New York

## Abstract

**Importance:**

HIV transmission rates in the United States have increased among men who have sex with men. However, there are no published randomized trials examining interventions to reduce sexual risk for HIV acquisition in males younger than 18 years.

**Objective:**

To determine the efficacy of MyPEEPS Mobile, a mobile-delivered HIV prevention intervention, to reduce sexual risk behavior in same-sex attracted young males.

**Design, Setting, and Participants:**

This was a national randomized clinical trial of the efficacy MyPEEPS Mobile vs a delayed intervention among males aged 13 to 18 years to prevent or reduce sexual risk for HIV acquisition. Study activities were completed through 4 study sites in Birmingham, Alabama; New York, New York; Seattle, Washington; and Chicago, Illinois. Study staff at each site met with participants in person or via video conferencing. Data were collected from June 1, 2018, to April 7, 2020, and analyzed from July to October 2021.

**Interventions:**

The MyPEEPS Mobile intervention contains 21 online psychoeducational and skill-building modules, which participants completed over a 3-month period. Participants randomized to the intervention group received access to MyPEEPS Mobile for the first 3 months, while those randomized to the delayed intervention group received access at their 9-month visit after data for the primary efficacy analysis had been collected.

**Main Outcomes and Measures:**

The self-reported primary outcome was change in the number of condomless anal sex acts between study conditions. Secondary outcomes were change in the number of sex partners, number of condomless anal sex partners, the number of sex acts while under the influence of substances, preexposure prophylactic uptake, nonoccupational postexposure prophylaxis use, and HIV and sexually transmitted infection testing.

**Results:**

In the analytic sample of 763 racially and ethnically diverse study participants, the mean (SD) age was 16.2 (1.4) years; 736 participants (97%) were male, 13 (2%) nonbinary; and 6 (1%) genderqueer; 158 (21%) were Black or African American, 311 (41%) were Hispanic or Latino, and 284 (37%) were White. Overall, 382 were randomized to the intervention group and 381 to the delayed intervention group. At 3-month follow-up, there was a significant reduction in the number of condomless anal sex acts in the intervention group compared with the delayed intervention group (incidence rate ratio [IRR], 0.56; 95% CI, 0.32-0.99); however, there was no significant difference between groups at 6 or 9 months. In subgroup analyses, the intervention effect was pronounced among Black non-Hispanic participants at 3-month follow-up (IRR, 0.19; 95% CI, 0.04-0.94) and 6-month follow-up (IRR, 0.15; 95% CI, 0.03-0.78) compared with the delayed intervention group. There were no significant differences in the change in the number of sex partners, number of condomless anal sex partners, the number of sex acts while under the influence of substances, preexposure prophylactic uptake, nonoccupational postexposure prophylaxis use, and HIV and sexually transmitted infection testing between the intervention and delayed intervention groups.

**Conclusions and Relevance:**

In this study, the MyPEEPS Mobile intervention demonstrated a 44% overall reduction in condomless anal sex at 3-month follow-up compared with the delayed intervention group, but not at 6 or 9 months. To our knowledge, MyPEEPS Mobile is the first intervention to demonstrate evidence of short-term efficacy for reducing sexual risk among same-sex attracted young males.

**Trial Registration:**

ClinicalTrials.gov Identifier: NCT03167606

## Key Points

**Question:**

Does the MyPEEPS Mobile intervention reduce condomless anal sex acts among same-sex attracted adolescent males?

**Findings:**

In this randomized clinical trial of 763 same-sex attracted adolescent males, individuals randomized to the MyPEEPS Mobile intervention had a significantly greater reduction in condomless anal sex acts compared with those in the delayed intervention group during the initial 3-month follow-up period; there was no significant difference at 6 or 9 months.

**Meaning:**

The MyPEEPS Mobile intervention reduced short-term sexual risk for HIV infection in same-sex attracted adolescent males, a population at high risk of HIV infection.

## Teaser

This randomized clinical trial evaluates the efficacy of MyPEEPS Mobile, a mobile-delivered HIV prevention intervention, on reducing the sexual risk behavior of same-sex attracted adolescent males.

## Introduction

Men who have sex with men (MSM) comprise approximately 2% of the US population yet represent more than half of persons with HIV (PWH), accounting for nearly 70% of new HIV infections annually.^1,2^ Among MSM, risk for HIV acquisition is not evenly distributed. Racial and ethnic minority MSM have the highest rates of new HIV infections,^3^ with Black MSM accounting for 26% of new HIV diagnoses in 2019.^4^ In a recent analysis, new infections increased by 20% among Latino MSM, with younger men at significantly higher risk for undiagnosed HIV infection.^5^ Regarding age, younger MSM are disproportionately affected by HIV, with 27% of new cases in the United States among Black MSM aged 13 to 24 years and 22% among Hispanic and Latino MSM.^6^ Contributing factors are psychosocial (eg, bullying, victimization, isolation), contextual (eg, family, peer, and partner relationships), and behavioral (eg, number of sexual partners, condom use, and testing for HIV and sexually transmitted infections [STIs]).^7^

Interventions among young MSM, prior to or around the time of sexual initiation, align with the national strategy to focus on HIV prevention with key populations at risk for HIV.^8^ This presents the opportunity to use mobile health (mHealth) technology, a powerful platform for delivering HIV prevention with the potential to transform how health care and health education are provided and consumed.^9,10^ High mobile phone penetration in the United States,^11^ especially among racial and ethnic minority groups and youth,^12^ creates the opportunity for portable health interventions with enhanced privacy.^13,14^ Evidence suggests that mHealth-based interventions are a salient and promising method to increase reach in key populations.^15,16^ A review of mHealth interventions for high-risk MSM found that web-based videos and educational modules reduced HIV risk behavior and promoted HIV testing.^17^ Among youth, evidence suggests web-based interactive and educational approaches are efficacious for delaying sexual initiation,^18,19^ increasing knowledge of HIV and STIs, and promoting condom self-efficacy.^20^

There is a need to develop and test the efficacy of HIV prevention interventions for diverse racial and ethnic populations of young MSM, especially those younger than 18 years. There remains a dearth of evidence-based interventions for diverse adolescent MSM. More than 3 decades into the HIV epidemic, the current US Centers for Disease Control and Prevention (CDC) compendium listing of evidence-based behavioral interventions (EBIs) for HIV prevention has none with demonstrated efficacy among MSM younger than 18 years, and none have been developed targeting diverse, multiethnic adolescent MSM.

In response to the lack of HIV prevention EBIs for adolescent MSM, our study team adapted MyPEEPS, a group-based HIV prevention curriculum, into MyPEEPS Mobile. MyPEEPS was developed as a group-based intervention for diverse young MSM ages 16 to 20 years.^21,22,23^ The intervention demonstrated efficacy reducing sexual risk in this population.^24^ We adapted the curriculum to a mobile app—MyPEEPS Mobile—for a younger (13-18 years) and diverse group (ie, inclusive of American Indian and Alaskan Native, Native Hawaiian, and Asian individuals) through a user-centered, iterative design process. MyPEEPS Mobile was tested for feasibility, acceptability, and usability.^25,26,27,28^ This study sought to answer the following research question: does the MyPEEPS Mobile intervention reduce condomless anal sex acts among same-sex attracted adolescent males?

## Methods

### Study Design and Participants

This study was a randomized clinical trial (RCT) of MyPEEPS Mobile vs delayed intervention on condomless anal sex acts among same-sex attracted adolescent males at 3, 6, and 9 months after baseline. It follows the Consolidated Standards of Reporting Trials (CONSORT) reporting guidelines. The trial protocol appears in Supplement 1. Recruitment was completed at study sites in Birmingham, Alabama; New York, New York; Seattle, Washington; and Chicago, Illinois. While our initial plan was to recruit youth locally for in-person visits, reaching local enrollment targets proved challenging, and therefore, enrollment procedures were expanded nationally to online platforms. While some recruitment took place at local community-based organizations, most participants were recruited via free and paid online national advertisements promoted on Reddit, Facebook, SnapChat, and Instagram. Study sites were responsible for enrolling participants within their regional area detailed in eFigure 1 in Supplement 2.

Eligibility criteria included (1) being aged 13 to 18 years; (2) being assigned male sex at birth and self-identify as male, nonbinary, and/or genderqueer; (3) being able to read English; (4) living in the United States and its territories; (5) owning or having access to a mobile device (eg, smartphone or tablet); (6) having a self-reported attraction to males and/or a history of sexual activity or interest to engage in sexual activity with other males in the next 12 months; and (7) being self-reported HIV negative or unknown status.

Columbia University served as the single institutional review board for all study activities.^29^ An independent data safety and monitoring board was convened for this study and met annually to monitor the study. Written or electronic informed assent (for participants <18 years) and consent (for participants aged 18 years) was obtained for participants with parental consent waived for minors. Upon enrollment, participants were required to provide photo identification with date of birth to confirm age and identity. Incentives were provided for study visits ($25 initial visit, $30 at 3 months, $35 at 6 months, and $40 at 9 months [participants randomized to the delayed intervention also received $45 for completing an assessment at 12 months]). Participants received an additional $100 for completing all MyPEEPS Mobile activities. Baseline visits were completed either in person or via Zoom video conferencing for participants enrolled remotely. Follow-up visits were conducted remotely, and participants completed a Qualtrics survey sent to them electronically.

### Intervention

MyPEEPS Mobile was based on an adapted social-personal theoretical framework for young MSM,^30^ building on social learning theory^31^ and focusing on psychosocial (eg, affect regulation) and contextual risk related to HIV risk among MSM (eg, family, peer, partner relationships). MyPEEPS Mobile provides educational information about HIV and STIs, raises awareness about minority stress,^32^ and builds skills for condom use, emotion regulation, communication between participants, their families, and potential partners.^24^ The learning process was facilitated through stories of 4 “peeps” (Philip, Nico, Artemio, and Tommy) who were composites of young MSM participating in the development of the original MyPEEPS intervention.^21,22^ A running theme throughout the intervention is sexual risk reduction and goal-setting through an activity called BottomLine in which participants set goals and are prompted to reconsider or refine these goals after exposure to intervention activities (ie, building knowledge, self-awareness, and self-efficacy). Content is delivered through games, scenarios, and role-plays within 21 mobile activities illustrated in eFigure 2 in Supplement 2.^28^ All content was accessible between randomization and the 3-month follow-up visit (ie, content did not expire and could be revisited) and had to be completed in a linear manner. Privacy was protected via log-in and password credentials and automatic log off after 20 minutes of inactivity.

### Randomization

We used block randomization, stratified by site, to assign participants to the intervention or delayed intervention group. Treatment assignment was predetermined and blinded, and assignment remained static throughout the course of the trial to reduce selection bias. While we did not stratify the sample by age or rural vs nonrural area, we monitored recruitment closely to promote inclusion of younger participants (ie, ages 13 and 14 years) and from rural-designated areas. We concealed randomization status from staff and participants until completion of the baseline assessment. Participants randomized to the intervention group received access to MyPEEPS Mobile for the first 3 months, while those randomized to the delayed intervention group received access at their 9-month visit after data for the primary efficacy analysis had been collected. Participants completed surveys every 3 months.

### Study Assessments

Participants completed standardized quantitative assessments of demographic characteristics (age, race and ethnicity, rural residency) and sexual behavior at baseline and 3-, 6- and 9-month follow-up visits via Qualtrics (those randomized to the delayed intervention received an additional assessment at 12 months). The primary outcome was change in the number of recent condomless anal sex acts (prior 3 months) on a modified version of the AIDS Risk Behavior Assessment.^33^ Sequential questions asked participants to estimate the number of recent anal sex partners (ie, insertive and receptive) and the number of condomless sex acts with partners, which provided the basis for the primary outcome (a count variable). In addition, satisfaction with the MyPEEPS Mobile intervention was assessed using the 8-item Client Satisfaction Questionnaire (CSQ-8)^34^ at 3 and 12 months in the intervention and delayed intervention groups, respectively.

### Statistical Analysis

We targeted enrollment of 700 participants with at least 70 participants in subgroups by age (ie, 13 years, 14 years), racial and ethnic group, and rural-designated areas. The primary power analysis was based on the main outcome (number of condomless anal sex acts with male partners in the past 3 months) in 2 scenarios: (1) overall effect with total participants and (2) stratified analysis by subgroup (age, racial and ethnicity, and rural areas). The following assumptions were used for the power estimation: (1) an 80% retention rate (analytic sample, 560 total and 56 for subgroups); (2) a conservative and high intracluster correlation of 0.8, due to repeated measured data of the same individuals; (3) mean number of recent condomless anal sex acts with male partners at baseline^35^ is 1.2; and (4) all power estimations were based on α = .05 and 2-sided tests. Findings from the prior MyPEEPS study indicated condomless anal sex acts decreased by 63% or a relative risk (RR) of 0.37.^35^ However, the large effect was not statistically significant. Because the estimated effect size of the intervention was unreliable, we used an RR of 0.73, 1 SE greater than the estimated RR of 0.37 to provide a conservative estimate for both overall and subgroup analyses. To examine the overall effect of the intervention accounting for 20% attrition, we estimated 97% power to detect a RR of 0.73 with analytic sample size of 700 participants. We also powered our study to conduct stratified analysis based on race and ethnicity and determined we would have 92% power to detect a relative risk of 0.37 in each racial and ethnic subgroup (eg, Black, non-Hispanic; Asian and Native Hawaiian or other Pacific Islander).

We used a generalized linear mixed models (GLMMs) with a negative binomial distribution for count variables (ie, condomless anal sex acts, number of sexual partners, number of condomless anal sex partners, and anal sex acts under the influence of substances) and binomial distribution for dichotomous variables (ie, preexposure prophylactic [PrEP] uptake, nonoccupational postexposure prophylaxis use, HIV testing, and STI testing) to examine the impact of the intervention on each outcome variable. To examine the difference in the rate of change for the outcome variables,^36^ we used mixed-effects models with a participant-level random intercept to allow the baseline outcome measure (eg, condomless sex acts) to vary across participants and account for within-participant correlation.

To measure MyPEEPS intervention efficacy in reducing condomless anal sex acts, we calculated interactions between study group (ie, delayed intervention vs intervention) and each indicator for time (ie, 3, 6, and 9 months) following the baseline observation, indicating a difference in the rate of change from baseline to each time across groups. Models controlled for race and ethnicity, age, and online or offline enrollment. This model was followed by a stratified analysis by race and ethnicity. Missing data for the primary outcome at each time point ranged from 16.7% (at 3 months) to 19.2% (at 9 months). For variables with a significant intervention effect, a secondary analysis was conducted to examine within-person change. Baseline and 3-month data were used for the intervention group, and 9- and 12-month data were used for the delayed intervention group. Baseline GLMMs examined change pre-post and a second model including an interaction between study group and pre-post change, which would indicate a variation in the effect of the intervention by group. Analyses were conducted in R version 4.0.4 (R Project for Statistical Computing).

## Results

### Sample Characteristics

The mean (SD) age of the 763 participants was 16.2 (1.4) years, with at least 70 participants in each age group except those aged 13 years, of whom 20 were enrolled. A total of 85 participants (11%) reported residing in rural-designated areas; 736 participants (97%) were male, 13 (2%) nonbinary; and 6 (1%) genderqueer; 284 (37%) identified as White, 158 (21%) as Black or African American, 72 (9%) as Asian, 43 (6%) as American Indian or Alaskan Native, and 11 (1%) as Native Hawaiian or other Pacific Islander. By ethnicity, 311 participants (41%) identified as Hispanic or Latino (any race). To try to enroll the minimum of 70 in each racial and ethnic, age, and rurality subgroup, we closed enrollment to certain age and racial and ethnic subgroups and participants from urban areas at different points during the study. Details on the open and closed enrollment groups appear in eTable 1 in Supplement 2.

**Figure 1.**
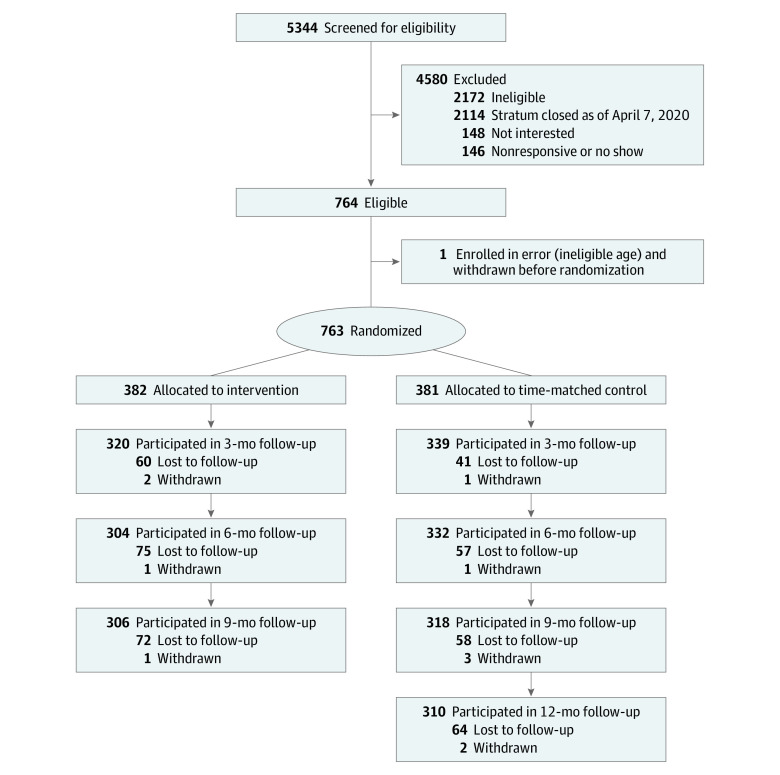
Study Flow Diagram

From June 1, 2018, to April 7, 2020, 5344 individuals were screened, 764 enrolled, and 763 randomly assigned to 2 cohorts: 382 individuals to the MyPEEPS Mobile intervention, and 381 to the delayed intervention arm (Figure 1). One participant was enrolled in error (ineligible age) and withdrawn prior to randomization. Most of the sample (606 [79%]), enrolled online. A total of 310 participants (41%) reported any (lifetime) condomless anal sex at baseline (Table 1). A total of 155 participants enrolled in person, with 22 (13%) in Chicago, 25 (26%) in Birmingham, 93 (27%) in New York, and 15 (11%) in Seattle.

**Table 1.** Characteristics of 763 Participants in the MyPEEPS Mobile Randomized Clinical Trial, Overall and by Study Condition

Characteristic^a^	No. (%)		
	Overall (n = 763)	MyPEEPS intervention (n = 382)	Delayed MyPEEPS intervention (n = 381)
Study site			
Birmingham, Alabama	98 (12.9)	48 (12.7)	50 (13.1)
Chicago, Illinois	175 (23.0)	87 (23.0)	88 (23.1)
New York City, New York	350 (46.1)	176 (46.4)	174 (45.7)
Seattle, Washington	137 (18.0)	68 (17.9)	69 (18.1)
Age, mean (SD), y	16.22 (1.4)	16.23 (1.4)	16.20 (1.4)
Gender identity			
Male	736 (97.0)	368 (97.1)	368 (96.8)
Genderqueer	6 (0.8)	4 (1.1)	2 (0.5)
Nonbinary	13 (1.7)	4 (1.1)	9 (2.4)
Other	4 (0.5)	3 (0.8)	1 (0.3)
Sexual orientation			
Only gay or homosexual	406 (53.5)	214 (56.5)	192 (50.5)
Mostly gay or homosexual	166 (21.9)	74 (19.5)	92 (24.2)
Bisexual	161 (21.2)	77 (20.3)	84 (22.1)
Mostly heterosexual	4 (0.5)	2 (0.5)	2 (0.5)
Only heterosexual	2 (0.3)	1 (0.3)	1 (0.3)
Other	20 (2.6)	11 (2.9)	9 (2.4)
Race			
American Indian or Alaskan Native	43 (5.7)	20 (5.3)	23 (6.0)
Asian	72 (9.5)	36 (9.5)	36 (9.5)
Black or African American	158 (20.8)	75 (19.8)	83 (21.8)
Native Hawaiian or other Pacific Islander	11 (1.5)	6 (1.6)	5 (1.3)
White	284 (37.4)	132 (34.8)	152 (39.9)
Multiracial	94 (12.4)	55 (14.5)	39 (10.2)
Missing or unknown	98 (12.9)	55 (14.5)	43 (11.3)
Ethnicity			
Hispanic or Latino	311 (40.9)	169 (44.6)	142 (37.3)
Not Hispanic or Latino	449 (59.1)	210 (55.4)	239 (62.7)
Born outside United States	54 (7.1)	28 (7.4)	26 (6.9)
Not a current student	29 (3.8)	12 (3.2)	17 (4.5)
Highest education level			
Sixth grade	1 (0.1)	1 (0.3)	0 (0.0)
Seventh grade	21 (2.8)	9 (2.4)	12 (3.2)
Eighth grade	53 (7.0)	25 (6.6)	28 (7.4)
Some high school	539 (71.3)	266 (70.4)	273 (72.2)
High school diploma/GED	92 (12.2)	47 (12.4)	45 (11.9)
Some college	50 (6.6)	30 (7.9)	20 (5.3)
Rural county of residence	85 (11.2)	41 (10.9)	44 (11.6)
No primary care clinician	206 (27.5)	96 (25.5)	110 (29.5)
Ever had HIV test	252 (33.6)	128 (34.0)	124 (33.2)
Any sexual activity with another male	517 (68.9)	263 (69.8)	254 (68.1)
Any anal sex acts with another male	395 (52.0)	203 (53.6)	192 (50.4)
Combined insertive and receptive anal sex partners within past 3 mo, median (IQR), No.	1 (0-2)	1 (0-2)	1 (0-2)

^a^ Data were missing as follows: born outside the United States, 3 participants; not a current student, 1 participant; highest education level, 4 participants; rural county of residence, 2 participants; no primary care clinician, 10 participants; ever had HIV test, 10 participants; any sexual activity with another male, 10 participants; and combined No. of insertive and receptive anal sex partners within past 3 months, 11 participants.

### Primary Outcome

**Figure 2.**
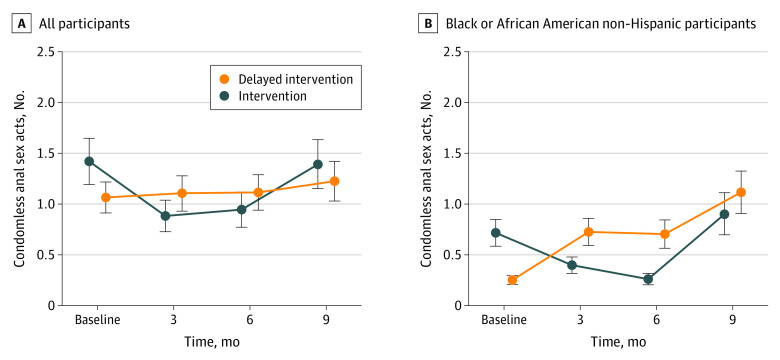
Mean Number of Condomless Sex Acts Whiskers indicate SDs.

The sample for analysis of intervention efficacy was 761. For condomless sex acts, we found a significantly different rate of change in the change in number of condomless sex acts (Table 2) in the intervention group from baseline to 3 months compared with the delayed intervention group (incidence rate ratio [IRR], 0.56; 95% CI, 0.32-0.99). Estimated means are presented in Table 3. Therefore, participants randomized to the MyPEEPS Mobile intervention group had a significant reduction in condomless anal sex acts compared with the delayed intervention arm at 3 months. However, there was no significant difference between the intervention and delayed intervention groups between baseline and 6 months or 9 months. For subgroup analyses, the intervention effect was especially pronounced and durable among Black or African American non-Hispanic participants (eTable 2 in Supplement 2), with a significantly different rate of change between baseline and 3 months (IRR, 0.19; 95% CI, 0.04-0.94) in the intervention group compared with the delayed intervention group and baseline and 6 months in the intervention group compared with the delayed intervention group (IRR, 0.15; 95% CI, 0.03-0.78), but the difference between baseline and 9 months was not statistically significant (Figure 2). The secondary analysis examining within-person change for all participants indicated that the intervention had a significant effect on condomless anal sex acts (IRR, 0.78; 95% CI, 0.62-0.99) in the number of condomless sex acts before the intervention (estimated mean [SE], 1.27 [0.14]) compared with after (estimated. mean [SE], 0.91 [0.07]). In the model that included an interaction between pre-post change and intervention group, there was no significant difference (IRR, 0.71; 95% CI, 0.44-1.14) in the effect of the intervention by group, so the original model was retained. For the other primary outcomes, there were no significant differences in the change in the number of sex partners, the number of condomless anal sex partners, the number of sex acts while under the influence of substances, PrEP use, HIV testing, or STI testing between the intervention and delayed intervention groups between baseline and 3 months, 6 months, or 9 months (Table 2).

**Table 2.** Multivariate Results for Primary Outcomes

Model^a^	IRR (95% CI)				OR (95% CI)			
	Condomless sex acts	No. of sex partners	No. of CAS partners	Sex acts using substances	PrEP Use	nPEP	HIV Testing	STI Testing
Time, IRR (95% CI)								
Baseline	[Reference]	[Reference]	[Reference]	[Reference]	[Reference]	[Reference]	[Reference]	[Reference]
3-mo	1.10 (0.74-1.63)	0.80 (0.58-1.09)	0.88 (0.60-1.28)	0.86 (0.51-1.46)	10.56 (1.91-58.44)	0.63 (0.11-3.72)	2.90 (1.77-4.76)	0.82 (0.48-1.41)
6-mo	1.04 (0.69-1.59)	0.99 (0.72-1.36)	1.18 (0.81-1.72)	1.09 (0.65-1.83)	17.99 (3.10-104.46)	2.71 (0.53-13.97)	2.47 (1.49-4.10)	0.76 (0.44-1.32)
9-mo	1.18 (0.79-1.77)	0.89 (0.65-1.22)	0.94 (0.64-1.38)	1.32 (0.79-2.20)	10.74 (1.88-61.40)	0.43 (0.06-2.85)	2.55 (1.53-4.24)	0.76 (0.44-1.32)
Intervention, IRR (95% CI)	1.56 (0.91-2.68)	1.10 (0.81-1.49)	1.27 (0.88-1.83)	0.72 (0.32-1.62)	0.36 (0.02-6.92)	0.56 (0.04-8.62)	1.18 (0.64-2.16)	1.16 (0.62-2.18)
Intervention × time, IRR (95% CI)								
×3-mo	0.56 (0.32-0.99)	1.16 (0.74-1.81)	1.09 (0.64-1.87)	0.91 (0.41-1.99)	1.65 (0.12-23.19)	1.81 (0.11-30.40)	1.18 (0.59-2.35)	1.45 (0.69-3.02)
×6-mo	0.61 (0.34-1.08)	0.99 (0.63-1.54)	0.73 (0.43-1.26)	0.64 (0.28-1.46)	5.31 (0.34-83.90)	1.34 (0.10-18.70)	1.28 (0.64-2.60)	1.26 (0.59-2.69)
×9-mo	0.83 (0.47-1.47)	1.20 (0.76-1.87)	1.27 (0.74-2.17)	1.51 (0.71-3.22)	3.70 (0.23-58.41)	0.76 (0.03-21.96)	1.72 (0.85-3.47)	1.54 (0.72-3.28)

Abbreviations: CAS, condomless anal sex; IRR, incidence rate ratio; nPEP, nonoccupational postexposure prophylaxis; PrEP, preexposure prophylaxis; STI, sexually transmitted infection.

^a^ All models controlled for race and ethnicity, age, study site, and recruitment method (ie, online or in person).

**Table 3.** Estimated Mean and SE for Condomless Sex Acts

Time	Estimated mean (SE)			
	All		Black or African American Non-Hispanic	
	Intervention	Delayed intervention	Intervention	Delayed intervention
Baseline	1.42 (0.23)	1.07 (0.15)	0.71 (0.13)	0.25 (0.04)
3-mo	0.88 (0.16)	1.10 (0.17)	0.40 (0.08)	0.72 (0.13)
6-mo	0.94 (0.17)	1.11 (0.17)	0.26 (0.06)	0.70 (0.14)
9-mo	1.39 (0.24)	1.22 (0.20)	0.90 (0.21)	1.12 (0.21)

Regarding feasibility of intervention delivery, 698 of 763 participants (91.5%) were given access to the app, and 570 (81.7%) completed all app modules. A total of 65 participants (8.5%) did not receive app access due to withdrawal from the study or loss to follow-up before receiving access. With respect to participant acceptability and satisfaction, of those who completed the CSQ-8,^34^ 605 of 623 (97.1%) rated MyPEEPS Mobile as good or excellent, and 585 (93.9%) indicated that they would probably recommend MyPEEPS Mobile to friends.

Retention rates for each study group remained at or greater than 80% throughout the study. There were no significant associations between participants being lost to follow-up and the number of condomless sex acts at baseline, any covariates at baseline, or study arm assignment in a GLMM binomial model.

## Discussion

The MyPEEPS Mobile intervention demonstrated a 44% reduction in condomless sex acts in the intervention group compared with the delayed intervention group at 3 months. In addition, 81% and 85% reductions in condomless anal sex acts were reported at 3 and 6 months, respectively, for Black or African American participants in the intervention group compared with the delayed intervention group. Collectively, these findings suggest a robust, short-term intervention effect. These intensified effects in Black participants are particularly salient because it is estimated that 50% of Black MSM will develop HIV in their lifetimes compared with 1 in 11 White MSM.^37^ Our findings provide evidence that MyPEEPS Mobile is a feasible, acceptable, and efficacious intervention that reduces HIV risk behavior among MSM aged 13 through 18 years.

To our knowledge, there have been few evidence-based HIV prevention interventions to date developed for adolescent MSM in comparison with other high-risk demographic groups (eg, adult MSM, women). Previous studies have been conducted with MSM as young as 15 years, but to our knowledge, this is one of the youngest study samples in the United States. Furthermore, our study sample was diverse, with more than 75% of study participants from racially or ethnically diverse backgrounds. These findings have important implications given that new HIV infections increased by 20% among Latino MSM in 2017,^5^ and 27% of new HIV cases in the US were among Black MSM aged 13 to 24 years.^6^ In the current study, only 52% of participants reported a history of anal sex; however, 78% of those participants reported having condomless anal sex. That we were able to intervene with youths, either before sexual initiation or early in their sexual trajectory, and demonstrate a reduction in HIV sexual transmission risk speaks to the feasibility and acceptability of targeting HIV prevention intervention before or around the time of sexual debut.

We also successfully demonstrated that an intervention originally designed as a group-based intervention can be translated to a mobile app and maintain efficacy. Given the much greater reach of mobile interventions in comparison with group-based modalities, this has important implications for this young target population. Therefore, findings from this study are worth noting in the context of their implications for delivery of behavioral health interventions. Mobile technology, given its ubiquity, offers an ideal platform for reaching people who may not be able or willing to come in person to receive critical health information.

Study participants only had access to the MyPEEPS Mobile app from baseline to 3 months. Given the attenuating effects of the intervention after 3 months for most participants, future evaluation of MyPEEPS Mobile should allow participants to have continued access to the app or integrate booster sessions and assess whether these provide for a more sustained intervention effect.

The CDC High Impact HIV/AIDS Prevention project^38^ publishes and continually updates a Compendium of HIV Prevention Interventions with Evidence of Effectiveness.^39^ Currently, this compendium includes 47 active HIV risk reduction, evidence-based behavioral interventions. At present, there is 1 evidence-based, mHealth intervention that targets HIV-negative MSM aged 18 to 29 years.^40^ However, to our knowledge, there are no interventions available that target adolescent same-sex attracted males aged 13 to 18 years. More interventions for diverse adolescent young MSM are needed that meet criteria outlined in the CDC’s Compendium for Best Evidence risk reduction.^39^ As an mHealth delivered intervention with a rigorous randomized clinical design, MyPEEPS Mobile meets these criteria.

Importantly, we struggled to recruit and enroll males aged 13 years as well as some racial groups, such as Native Hawaiian and other Pacific Islander individuals. Future work should seek to understand the barriers to enrolling these same-sex attracted adolescents. Consideration of whether these interventions are relevant to young participants is warranted. Furthermore, it is important to evaluate whether these younger adolescents can access recruitment material and intervention content given differences in social factors, such as the structure, hours, and activities in elementary vs high school and parental involvement and/or monitoring for adolescents aged 13 years. Developmental reasons, including potentially a limited number of early adolescents who may have been “out” about their sexual orientation, may have also contributed to early adolescents’ unwillingness or inability to participate in this study. Finally, community-based approaches are needed for reaching and targeting recruitment materials specifically to the needs of Native Hawaiian and other Pacific Islander communities.

### Limitations

This study has limitations that should be considered when interpreting these findings. This study recruited participants with smartphone or smart device access using advertisements on popular social media; thus, findings cannot be generalized to those without access to smart devices and/or social media platforms. Selection bias is an additional limitation, and there is a need to understand characteristics of age groups willing to engage in HIV prevention activities. Furthermore, self-reported outcomes introduce recall and social desirability bias especially given the duration of time between result ascertainment. While this study fills a gap in the prevention intervention science, findings are limited to behavioral effects of the intervention on sexual risk rather than on HIV seroconversion. Future work should consider intervention effects on HIV incidence.

## Conclusions

Using the CDC compendium criteria^39^ as a framework, these findings suggest that MyPEEPS Mobile is a well-supported, evidence-based, behavioral risk-reduction intervention for HIV prevention among same-sex attracted adolescent males. Additional research is needed to replicate these findings and to assess the efficacy of MyPEEPS Mobile for reducing HIV incidence.

## Notes

Supplement 1.
Trial Protocol

Supplement 2. **eTable 1.** Open and Closed Enrollment Time-Points **eTable 2.** Stratified Analysis of Condomless Sex Acts for Black or African American Non-Hispanic Participants **eFigure 1.** MyPEEPS Enrollment by Region **eFigure 2.** Sample MyPEEPS Content and Activities

Supplement 3.
Data Sharing Statement
