# Supplementary material for: Efficacy of MyPEEPS Mobile, an HIV Prevention Intervention Using Mobile Technology, on Reducing Sexual Risk Among Same-Sex Attracted Adolescent Males: A Randomized Clinical Trial
Source: JAMA Netw Open. 2022 Sep 21;5(9):e2231853. doi: 10.1001/jamanetworkopen.2022.31853 (PMC9494195; doi:10.1001/jamanetworkopen.2022.31853)
Supplement: Supplement 1. — Trial Protocol [file jamanetwopen-e2231853-s001.pdf]

Title: **A Pragmatic Clinical Trial of MyPEEPS Mobile to Improve HIV Prevention Behaviors in Diverse Adolescent MSM**

NCT Number: **NCT03167606**

Document Date: **8/12/2022**

Table of Contents

A. Study Purpose and Rationale 4
A.1. Study Design 5
A.1.2. Study Sites. 5
A.2. Adaption of the MyPEEPS intervention to a mobile platform. 7
A.3. Participant inclusion and exclusion criteria for all Aims. 9
B. Study Aims 10
B.1. Specific Aim 1: Using qualitative methodology and usability assessments, update and translate the MyPEEPS intervention into MyPEEPS Mobile for diverse YMSM. 10
B.1.2. In-Depth Interviews with YMSM 11
B.1.3. Development of mobile delivery technology. 12
B.1.4. Refinement of the MyPEEPS Mobile user interface. 12
B.1.4.a Heuristic Evaluation. 13
B.1.4.b. End-User Usability Testing. 13
B.2. Specific Aim 2: Pilot test MyPEEPS Mobile in a sample of 40 YMSM (10 at each study site). 14
B.2.1. Pilot Study Cohort: 14
B.2.2. Screening: 14
B.2.3. Written Informed Consent Process for Study Participation. 14
B.2.4. Secure Web-based Baseline Assessment. 15
B.2.5. Intervention Delivery. 15
B.2.6. Secure Web-based Follow-Up Assessment. 16
B.2.7. Young Adult Pilot: Pilot Test MyPEEPS with 19-25 year old MSM (n=10 at each of 2 sites: NYC & Chicago) 16
B.2.8. Multi-site Focus Groups: Conduct Eight Sessions with Female Assigned at Birth (FAB) Trans Masculine & Gender Non-Conforming Youth, 13-18 Years Old, to Assess the Relevance and Adaptability of MyPEEPS Mobile 16
B.2.9. Pilot test a MyPEEPS Art Contest to Involve Native and Indigenous Youth in the Design of MyPEEPS Mobile recruitment material. 18
C. Specific Aim 3: Conduct a randomized 2-group multi-site RCT to evaluate the efficacy of MyPEEPS Mobile intervention to reduce HIV risk behaviors in 900 YMSM at 4 study sites. 19
C.1. Overview of the Study Design. 19
C.2. Sampling approach to successfully enroll YMSM participants in this national project. 20
C.3. Procedures for Maximizing Research Integrity. 20
C.4. Description of study population. 20
C.5. Sample Size and Power Calculation. 20
C.6. Sampling Approach. 21
C.7. Recruitment Approach. 21
C.7.a. Recruitment Venues. 21
C.7.b. Potential Problems/Alternative Solutions Regarding Recruitment: 23
C.8. Online Study Procedures 23
C.8.1. Eligibility screening. 24

C.8.1.a. Secondary Screening to Confirm Eligibility 24
C.8.2. Informed assent/consent. 24
C.8.3. Randomization to intervention and delayed intervention study arms. 25
C.9. Overview of data collection time points. 25
C.10. Design Considerations. 26
C.11. Study Outcomes. 27
C.11.a. Primary Outcome Measures. 27
C.11.b. 27
Secondary Outcomes. 27
C.11.c. Intermediate Outcome Measures. 27
C.12. COVID-19 Measures. 27
C.13. Sound Retention Efforts. 28
C.14. Statistical Procedures: 28
C.14.a. Missing data. 29
C.15. MyPEEPS Mobile RCT In-depth Interviews 30
D. Assess the feasibility of using In-home HIV tests for assessing HIV status in a sample of very YMSM by conducting follow-up testing with MyPEEPS RCT participants. 31
REFERENCES 33

A. Study Purpose and Rationale

HIV infection remains a significant public health problem especially among men who have sex with men (MSM). While MSM account for only about 2% of the U.S. population[1], they are the risk group most affected by HIV, constituting 56% of persons living with HIV.[2] Much of the increased incidence in HIV has been reported among young MSM (YMSM) and is linked to high-risk sexual behavior.[3] Disparities in HIV incidence exist among racial and ethnic minority groups and are particularly pervasive among younger MSM.[4]

At the same time, there remains a dearth of evidence-based HIV prevention interventions for diverse YMSM. To address this need, our study, in response to RFA-MD-15-012, leverages mobile technology and MyPEEPS, an existing theory-driven, multi-ethnic, group-level, evidence-based intervention for diverse YMSM.[5] MyPEEPS (R34MH079707; PI: Garofalo) is a manualized curriculum developed by members of our proposed Investigative team comprised of 6 modules focusing on key intermediate social and personal factors, including knowledge (e.g., correct way to use a condom), self-efficacy for safer sex, interpersonal communication skills and behavioral skills (Appendix A). MyPEEPS was tested with 101 diverse (23% White, 39% Black, 27% Latino, 12% other) YMSM, ages 16-20 and demonstrated evidence of feasibility, acceptability, and preliminary efficacy in reducing sexual risk behaviors.[5] As such, MyPEEPS is an ideal choice for use in this RFA as it is one of very few interventions in the literature targeted for diverse YMSM including those under 18 years of age.

Long-term sustainability of face-to face, group-level behavioral interventions, such as MyPEEPS, have been problematic for dissemination in at-risk populations, particularly among young racial and ethnic minority groups. In the initial MyPEEPS pilot trial, participants reported that a key difficulty with the intervention was the travel distance to access the group-based intervention.[5] In response to this challenge, we propose to translate MyPEEPS from a face-to-face, group-based curriculum to a mobile responsive driven web-based platform, accessible by smartphone or other web-enabled devices, to increase accessibility and scalability for diverse YMSM. The ubiquitous nature of mobile phones in daily life, especially among 13-18 year olds,[6] has created opportunities for health interventions in a portable format with enhanced privacy. mHealth approaches to HIV prevention intervention have demonstrated great promise to address sexual risk behaviors, promote sexual health and to optimize reach to those whose circumstances will not allow for in person attendance. By leveraging mobile technology, the proposed MyPEEPS Mobile intervention will deliver an HIV behavioral intervention to diverse YMSM to: 1) reach high-risk YMSM at a relatively low cost,[7-9] 2) engage YMSM where they meet sex partners,[10] and 3) enable YMSM to participate privately on a computer, tablet, or Smartphone on their own schedule, as opposed to in a structured setting.[11]

Using a participatory approach, our study will incorporate user-centered design in the translation of the MyPEEPS intervention onto a mobile platform. MyPEEPS was tested with older adolescents (16-18 year olds) and prior to the availability of non-occupational post-exposure prophylaxis (nPEP) and pre-exposure prophylaxis (PrEP); therefore, in addition to the mobile adaptation, we will update the intervention content in Aim 1. MyPEEPS Mobile will be delivered through YMSM avatars (e.g., caricature or graphic identity), on a mobile platform, whose profiles and problems are based on the formative research of the original pilot trial as well as the formative work proposed herein, and who manage their sexual health against a backdrop of personal, family-based, and relational challenges. MyPEEPS Mobile will be tested in an RCT with racially and ethnically diverse HIV-negative or unknown status YMSM aged 13-18 at four sites: Birmingham (AL), Chicago, New York City, and Seattle. Building upon our teams' extensive experience in HIV prevention, mHealth, behavioral interventions, and randomized controlled trials,[12-41] *the specific aims are to*:

1) Ensure acceptability of MyPEEPS across diverse groups and translate the MyPEEPS intervention into a mobile-based intervention for diverse YMSM (13-18 years).
2) Pilot test MyPEEPS Mobile in a sample of 40 YMSM (10 at each study site).
3) Conduct a randomized 2-group (intervention, control) multi-site trial, with randomization to two groups at each site, to evaluate the efficacy of MyPEEPS Mobile in reducing male-male sexual risk behaviors. Our primary outcomes will be: total number of sex partners and condomless anal sex partners, frequencies for condomless anal sex acts, anal sex with and without condoms under the

influence of alcohol/drugs, nPEP and PrEP (if FDA- approved for this age group) use and self-reported HIV/STI testing.

The proposed MyPEEPS Mobile intervention is a novel and evidence-driven intervention using mobile technology to deliver HIV prevention information specifically developed for at-risk YMSM. This will be the first study to test the efficacy of a scaled-up, mobile version of an existing HIV prevention intervention originally developed for, designed by, and piloted for, a diverse group of YMSM. If efficacious, this behavioral HIV prevention mobile intervention supports widespread dissemination.

In Summary, this application is significant because: 1) YMSM are at very high risk for acquiring HIV, compared to older MSM; 2) HIV disproportionately affects racial and ethnic minority MSM, though no HIV prevention initiatives have addressed diverse racial and ethnic minority YMSM; 3) MyPEEPS pilot data demonstrate evidence of feasibility, acceptability, and preliminary efficacy in reducing sexual risk behaviors. 4) eHealth interventions show efficacy in adult MSM but have not been developed for or tested in adolescents; 5) given the ubiquity and convenience of mobile technology, mobile platforms are well- suited to support behavioral interventions and health promotion targeting young populations; and 6) based on the aforementioned significance points on the need to conduct HIV prevention with YMSM, and after careful review of the original content of MyPEEPS, we believe that it is ideally suited for adaptation to a mobile platform. mHealth interventions for HIV risk reduction may be efficacious for youth, in general, and high risk populations more specifically,[42] and presents a unique opportunity to reach YMSM organically, as part of their everyday lives, with their personal devices and to disseminate information quickly and broadly.

A.1. Study Design

Overview. The proposed study is a multisite, mobile technology intervention of 900 HIV-negative or unknown status YMSM aged 13-18 from four U.S. cities (NYC, Chicago, Birmingham (AL), Seattle: RFA requirement to include participants from different US regions). Participants will be recruited both online and offline through social and sexual networking websites and apps, and through local CBOs, health centers, schools, and local events. The proposed study will translate an existing manualized group-based intervention, MyPEEPS, onto a mobile platform. Consistent with the design of the original MyPEEPS pilot, avatars will guide participants through the intervention components over a 3-month period. Our **Primary Outcomes for this study will be:** total number of sex partners and condomless anal sex partners, frequencies for condomless anal sex acts, anal sex with and without condoms under the influence of alcohol/drugs, nPEP and PrEP (if FDA approved for this age group) use and self-reported HIV/STI testing.

A.1.2. Study Sites.

Each of our sites has the experience and capacity to recruit the specified number of participants for this study. Our study sites have strong links to YMSM who are most affected and at-risk for HIV, demonstrating their ability to successfully recruit the target number of participants.[43-45] We have partnered with local scholars in HIV prevention research as well as community based organizations that have experience in providing services to YMSM (RFA requirement for relevant stakeholders). These local collaborations will allow us to meet our sample size requirements for the multi-site RCT, including adequate recruitment of racial/ethnic subgroups for analysis, and maximize generalizability to practice. A brief snapshot of each of the geographic locations is presented below. Further details about each site are included in the Facilities and Resources Section, subcontracts, letters of support from each site, and biosketches.

New York City (NYC) (Northeast) (Schnall, PI). Approximately 8.5 million people live in the 5 boroughs of NYC, with 1.4 million people living in the Bronx, 1.6 million in Manhattan and 2.6 million in Brooklyn. The racial/ ethnic breakdown across all 5 boroughs is: 44% White, 25.5% Black, 0.7% American Indian/ Alaskan Native and 12.7% Asian, and 0.1% Native Hawaiian or Pacific Islander.[51] In 2013, there were 117,618 persons living with HIV in NYC. Of these, 43,940 were infected through male to male sexual contact. Among those infected through male to male sex, the racial/ ethnic breakdown is: 30.4% Black, 37.5% White, 28.4% Latino, 2.5% Asian/ Pacific Islander, and 0.2%

American Indian and Alaskan Native.[46] We have a number of sites within NYC from where we will be recruiting. First, we have partnered with Callen- Lorde Community Health Center (CLCHC; see letter of support and subcontract), located across 3 clinical sites and 2 administrative sites in lower Manhattan and the Bronx. Its main clinical site is a 27,000-square foot, six- story building that is an ADA-compliant, fully licensed New York State Department of Health Article 28 Diagnostic and Treatment Center. In 2002, CLCHC was also designated a Federally Qualified Health Center.
Dr. Asa Radix will be leading the recruitment efforts at CLCHC. Of note, CLCHC has a location in the Bronx which is the hardest hit area of the HIV/AIDS epidemic in the country. The Bronx is one of the most underserved and poorest areas in the US with 38% of its residents living below the poverty line.[47] The Bronx has the highest HIV infection rate and death rate from HIV and is higher than in the rest of NYC. In fact, nearly 1 in 4 Bronx residents find out they are HIV-positive, find out the same day that they have AIDS.[48] The Health Outreach to Teens (HOTT) Program is the adolescent medicine program at CLCHC. Since 1989, The HOTT program has offered a vital link to health care and related services for LGBT and questioning youth. In 2015, the HOTT program had 1415 active patients between 13 and 24 years of age. Currently there are 157 male patients, ages 13-18 years, registered in HOTT. This age group is racially and ethnically diverse; 37% African American, 29% white, 12% multiracial, 6% Asian, 22% unreported; 34% Hispanic. The majority of HIV-negative youth at CLCHC are at risk for HIV and STIs. 30-40% engages in exchange of sex for money or shelter. Approximately 70% of HOTT clients use one or more illicit substances, e.g. heroin, crystal methamphetamine, cocaine or marijuana. Psychosocial issues facing the target population include: homelessness or unstable housing; history of violence or abuse; mental illnesses (such as depression, suicidal ideation, feelings of isolation and inadequacy); alcohol/drug abuse; unsafe sex; and involvement with the criminal justice system.

In addition to CLCHC, we have partnered with other community-based organizations and healthcare organizations in NYC. These include: APICHA, serving mainly Asian and Pacific Islander MSM, the LGBT Center in lower Manhattan, Ali Forney providing services for homeless YMSM, the NYC Department of Health and Mental Hygiene 8 STD Clinics, and Broome Street Academy (a high school for students with histories of homelessness/unstable housing, and foster care) (See Letters of Support).

Chicago, IL (Midwest) (Garofalo, MPI). There are approximately 5.2 million people living in Cook County with 2.7 million persons living in Chicago. The racial/ethnic breakdown of persons living in Chicago is: 45% White, 32.9% Black, 0.5% American Indian/ Alaskan Native and 0.5% Asian.[49] The 2013 rate of HIV infection diagnoses in Chicago (40.4 per 100,000) is approximately 2.5 times higher than the national rate, and the prevalence rate for Chicago (827.9/100,000) is nearly 3 times the national rate. In 2013, 22,346 persons in Chicago were living with HIV. Of those 13,292 were infected through male sex with male. Among those infected through male to male sex, the racial/ ethnic breakdown is: 38.6% Black, 35.5% White, 19.3% Latino, 1.2% Asian/ Pacific Islander, and <1% American Indian and Alaskan Native.[50] Our main recruitment site will be through The Center for Gender, Sexuality and HIV Prevention at Lurie Children's within the Division of Adolescent Medicine, directed by Dr. Garofalo. Additionally, we will advertise the study to the entire hospital system in the Chicago area to recruit younger adolescent YMSM and collaborate with community partners who serve very YMSM, ages 18 and younger in both schools and community-based environments, including Peer Health Exchange, The Illinois Caucus for Adolescent Heath, the Illinois Safe Schools Alliance, and the Broadway Youth Center of Howard Brown Health Center (See Letters of Support).

Birmingham, AL (South) (Mugavero, Site PI). There are approximately 212,000 people living in Birmingham, AL. The racial/ ethnic breakdown of persons living in Birmingham is: 22.3% White, 73.4% Black, 0.2% American Indian/ Alaskan Native and 1.0% Asian. During 2013, over half (55%) of the newly diagnosed cases and 43% of the prevalent cases reported male-to-male sexual activity as the primary risk factor for infection. The rate of HIV diagnosis among Black males is 6.8 times higher than White males in this geographic area.[51] We have partnered with Birmingham AIDS Outreach (BAO), a community based organization that has a number of innovative efforts to engage and provide service

for high-risk YMSM, such as the Youth Advisory Council, the Unicorn Pizza Club and the Magic City Acceptance Center. In addition, Advocates for Youth, a national advocacy and service organization that does extensive HIV prevention work with YMSM will use grassroots networks specifically working with AIDS Alabama in Birmingham to assist with recruitment at this site. The UAB CFAR (Co-Director Mugavero) will also be assisting with recruitment efforts. The UAB CFAR has successfully recruited from this study population in and BAO, along with the Alabama Department of Public Health, with Dr. Mugavero serving as site PI at UAB.

Seattle, WA (West) (Pearson, Site PI). There are approximately 2 million people living in Kings County with about 670,000 persons living in the City of Seattle. The racial/ ethnic breakdown of persons living in Seattle is: 69.5% White, 7.9% Black, 0.8% American Indian/ Alaskan Native and 13.8% Asian.[52] In 2015, there were 4,764 persons living with HIV in Seattle and 7,205 persons living with HIV in all of Kings County (including Seattle). Of those living with HIV, 67% were infected through male to male sexual contact. Among those infected, the racial/ ethnic breakdown is: 61% White, 19% Black, 13% Latino, 4% Asian, 1% Native American/ Alaskan Native and <1% Pacific Islander/ Native Hawaiian.[53] Seattle's lesbian, gay, bisexual, transgender and queer (LGBTQ) youth are disproportionately affected by homelessness with 22% of King County homeless people ages 12-25 are LGBTQ,[54] making Seattle an important site for recruiting homeless YMSM. Our main recruitment sites will be through the following community based organizations: Lambert House, Chief Seattle Club, Gay City and Seattle Children's Hospital (see letters of support). Dr. David Breland (consultant) will also assist with recruitment efforts. He is an adolescent medicine physician at Seattle Children's - the largest adolescent medicine program in the city and on the Board of Directors of local Seattle organizations serving the proposed target populations - thus well-poised to assist Co-I Pearson with recruitment activities in Seattle. The UW CFAR Community Action Board will also be involved in this project and will provide support for building additional collaborations between Drs. Pearson, Breland and community members and community-based organizations.

Importantly, the Seattle site is supported by the Indigenous Wellness Research Institute National Center of Excellence (IWRI NCE). In 2005, IWRI (www.iwri.org) was established under the UW 's Global Health Initiative with the mandate to nurture, develop, and advance a university-wide interdisciplinary indigenous research institute for health and health disparities research, knowledge sharing, and research capacity building with indigenous populations. IWRI NCE has an outstanding reputation among tribes and tribal organizations. For example, IW RI NCE partnered with the National Congress of American Indians (NCAI), a leading organization for tribal policy development and advocacy at the federal level representing over 250 federally-recognized tribes. Native community support is evident in all of our research and training initiatives.

In summary, our study sites have been funded by the CDC, SAMSHA, HRSA, NIH, private, local and State funding sources. The on-site investigators have led projects and have recruited from racially and ethnically diverse YMSM communities. We have established formal partnerships with our community based partners and academic sites, which demonstrates our commitment to working together, our shared vision for improving the risk behaviors and health outcomes in YMSM from diverse communities of color. To ensure a smooth collaboration, research team and CBO partners from each site will participate in an in person meeting in Year 1 of the study. The meeting will include a review of the current MyPEEPS intervention (See Aim 1) to identify areas in need of cultural adaption to ensure that MyPEEPS Mobile is acceptable across racial/ethnic groups. The meeting will follow with research team on monthly web-ex meetings or more frequent if needed. Each year, Drs. Schnall or Garofalo will travel to the other study locations (Seattle or Birmingham).

A.2. Adaption of the MyPEEPS intervention to a mobile platform.

Our goal in this project is to update and translate the current MyPEEPS intervention content into a mobile delivered intervention via a web app. The current MyPEEPS content, components and how it will be adapted is included in Appendix A. Table 1 is also a sample of the current content and how it will be adapted to a mobile platform. Each of the sessions from the original MyPEEPS will be converted into a module which will be comprised of a number of components to operationalize the existing MyPEEPS curriculum. To illustrate MyPEEPS Mobile will include didactic content, interactive quizzes,

graphical reports, videos, checklists, and to do list tools. The home screen of MyPEEPS Mobile will have a menu of the 6 modules that will need to be completed in consecutive order. We estimate that each module will take 60-90 minutes to complete. Participants do not need to complete a module in a single sitting.

Table 1. MyPEEPS adaption to a mobile platform

Session	Activities	Original Purpose	Components	Adaption to mobile platform
**Session 1: Logging on**	Introductions	To introduce participants to the program.	Components: Intro, underwear personality quiz	- Underwear personality: We will include a database of responses from others in our past studies as template responses for this activity.
**Session 2: HIV Knowledge**	HIV/STIs	To illustrate which sexual activities carry which risks, how to reduce those risks, and what symptoms to look for when it comes to getting tested and treated for STDs.	The basics about HIV/AIDS, STD symptoms, safer sex activity, “well-hung” (comparing high risk-low risk behaviors).	- Embed questions and allow them to answer via multiple choice or true false. Matching game: transmission modes to fluids and exchange.<br>- Game for correct condom steps - Adapt well hung for mobile using same symbols, hanging on clothes line.
**Session 3: Dealing with Stigma and Shame**	Dealing with Stigma	To familiarize participants with stigma management strategies and practice using these strategies with a partner	4 ways to deal with Stigma role- play	Go over stigma strategies and adapt role play exercise for mobile, identify advantages and disadvantages of styles and how this may impact sexual health.
**Session 4: From the Inside Out**	The ABCs of feelings	To illustrate how intense emotions influence behavior	ABCs of Triggers Triggering People, Places & Things	Pick a scenario and describe how they would react. Choose their triggering people places and things.
**Session 5: From the Outside In**	Influential Peeps	To highlight for participants the influential role of peers, family, and sexual partners on protective and risk behavior	Who are your influential BFFs? What’s Trust Got to Do With It?	- Influential BFFS: Animate calling out of important people and ask PT to add, examples of Boys who influence them,<br>- Elicit examples of when trust comes in to play for dating/sex, how we operate (rules) for protection
**Session 6: Logging Off**	Condom Use Review	To review for participants the basic condom skills covered in session two, and to reinforce/reiterate the importance of consistent and correct condom use	“Rubber Mis-hap”	Recreate rubber mishap through the use of YouTube videos or Vimeo.

We have partnered with Little Green Software (LGS) to create the mobile platform via a modular system. The modular system[55] will allow us to modify the intervention content after initial deployment as future efficacious behavioral intervention components are identified. As technology is rapidly changing, it is important to use a flexible development environment so that rapid changes can be made to the intervention content at a relatively low cost. Our mobile system will be developed as a web-app. Using responsive web design, the conventional web site is viewable on small screens and works well with touch screens. On a smartphone, a web-app appears and functions very similarly to a native app to the end-user. A web-app allows for media content that could not be included in a native app and is flexible for use across multiple devices.

Summary of Mobile Adaptation. After careful review of the existing MyPEEPS curriculum we believe it to be ideally suited for mobile adaptation. The mobile intervention will simulate a group-based “feel” including “mock” participants and comparison of participant responses to those of other YMSM.

Figure 1. Screenshot of MyPEEPS Mobile Module #4. Activity: The ABCs of Feelings

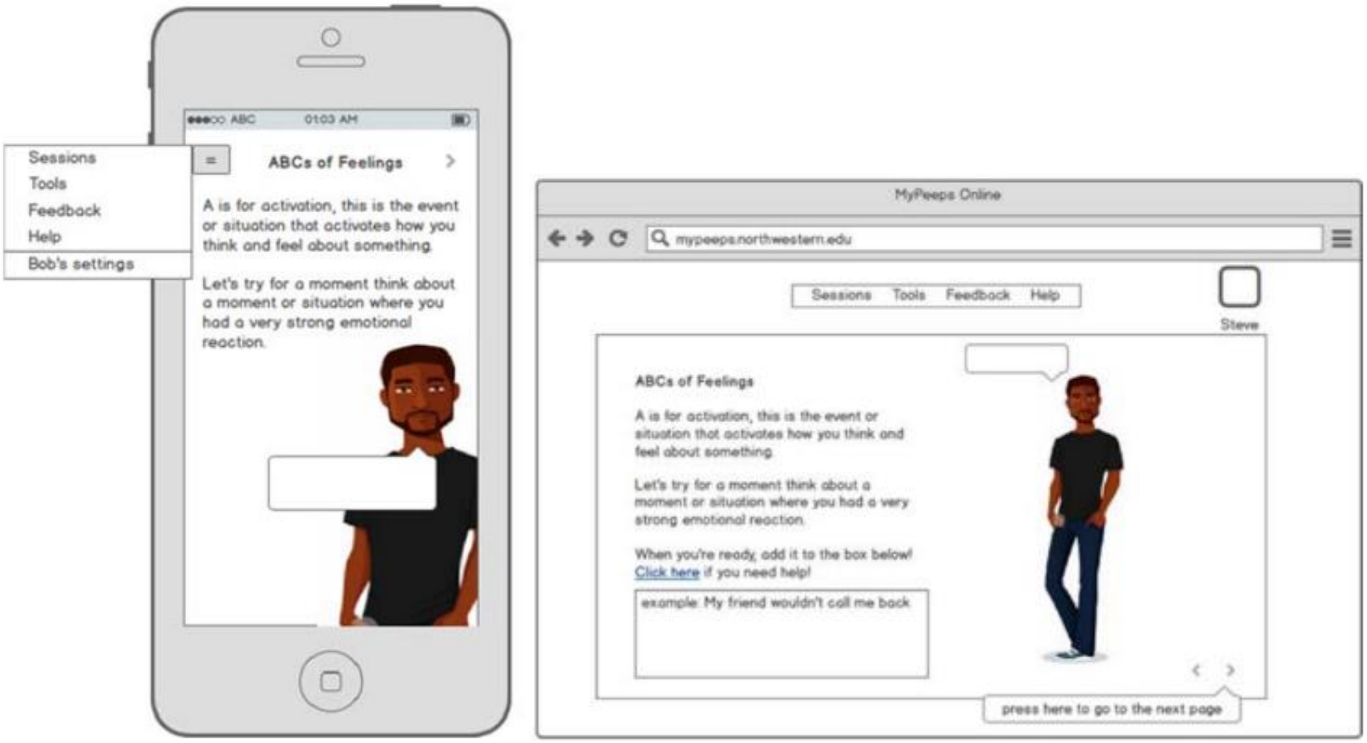

The learning activities in each session will be adapted to mobile format, using automated responses, drop-down menus, videos and games to engage the participant in the learning activities. Consistent with the original curriculum, the mobile Much like the current group- based in-person version of MyPEEPS, these characters will present scenarios to open each session and end with a “cliff hanger” to engage participants and motivate participation in the next session. The team at LGS has worked with members of our investigative team, (Drs. Schnall, Kuhns, Hidalgo) to develop the vision for the technical components of this intervention (see Appendix A for examples of mobile adaptation approaches for activities in each module and sample screenshots). A team of software engineers and project managers at LGS will perform the software development that will be required for this study. LGS is uniquely qualified to build the technical components of the system. We anticipate that MyPEEPS Mobile will be novel, innovative, scientifically sound, scalable, translatable, and highly acceptable; thus, it is likely to have a strong public health impact.

A.3. Participant inclusion and exclusion criteria for all Aims.

To participate in any aspect of the study, participants must be: between 13 and 18 years of age; self-identify as male, non-binary, and/or genderqueer; male sex assigned at birth; understand and read English; live in the US and its territories; own or have access to a mobile device (e.g. smartphone or tablet); self-reported attraction to males and/or a history of sexual activity or interest to engage in sexual activity with other males in the next 12 months; and self-report HIV-negative or unknown status.

We carefully considered the inclusion of transgender youth or YMSM that identify along the transfeminine spectrum but decided against it based upon our extensive clinical and research experience with this population. The underlying mechanisms of risk that lead to the acquisition of HIV are considerably different for transgender youth in comparison to the YMSM age 13-18 that this RFA seeks to target.[56] As one example, the literature on HIV acquisition and risk among young transgender women suggests that engagement in commercial sexual acts is a leading factor for the acquisition of HIV. In one study, young transgender women ages 16-24 with a history of engagement of commercial sex had a 23% prevalence of HIV versus 6% among those without a history of commercial sex

engagement. This risk factor is far less prevalent among YMSM. More to the point, the construct of commercial sex for transgender youth is complex in that it may affirm a gender identity for youth who often have difficulty identifying sexual or romantic partners as well as providing strong financial incentives (particularly for condomless anal sex) for a population of young people who face extreme degrees of economic marginalization.[57] Simply stated, YMSM ages 13-18 do not experience the same social forces. As such, we do not believe MyPEEPS Mobile (or any behavioral intervention) can be adequately culturally responsive to the HIV prevention needs of both adolescent YMSM and young transgender women or those who identify along the trans-feminine spectrum.[58-61]

Participant exclusion criteria. Males are ineligible to participate in the trial if: 1) they are HIV positive; 2) investigators determine that participation may be detrimental to the participant or to the study (e.g., severe cognitive deficit); or 3) they participated in the pilot phase of the project.

B. Study Aims

B.1. Specific Aim 1: Using qualitative methodology and usability assessments, update and translate the MyPEEPS intervention into MyPEEPS Mobile for diverse YMSM.

MyPEEPS was built on considerable formative work with multi- ethnic groups (R34MH079707; PI:Garofalo). Pilot findings have been peer reviewed and published.[62] We will continue to honor cultural difference while identifying common themes, concerns, and HIV risk and protective factors expand MyPEEPS and ensure its' acceptably as a multi-ethnic intervention. This will be done through reviews and continual feedback by our expert panels' and though focus groups from our end-users. This formative work focuses on translating the MyPEEPS intervention onto a mobile platform.

The goal of this Aim is to translate the information from the current curriculum into an interactive mobile app which will include the following components: games, True/False and multiple choice quizzes, videos. Using a community-based participatory approach, we will create the user interface for MyPEEPS Mobile through participatory design sessions and usability evaluations. Schnall and Pearson have successfully used participatory design sessions in developing mHealth interventions for culturally diverse groups.[16,17,19]

Purpose: The goal of this panel is to validate language, images, formatting/visual cues, and examples and reach censuses on common concerns, risk and protective factors.

Procedures: At the start of our study, we will hold a 1 day virtual meeting with the research team to review the curriculum, highlight areas where panel members' feedback will be most beneficial, and to review project timeline and deliverables. Two weeks after the virtual meeting, we will hold a 2 day in person and community leader expert panel members listed in Table 2. Experts or Co-Is who are not able to travel for the in-person meeting will participate via WebEx.

Table 2. Expert Review Panel Members

	Member	Title	Expertise
1	Jose Bauermeister MPH, PhD	Associate Professor of Health Behavior and Health Education, School of Public Health, University of Michigan	Latino YMSM
2	Bobbie Berkowitz PhD, RN, FAAN	Mary O'Neil Mundinger Professor of ursing Dean of Columbia University School of Nursing	LGBT Health
3	David Breland, MD, MPH	Associate Professor of Pediatrics, Clinical Director, Division of Adolescent Medicine, Seattle Children's	Black YMSM
4	Tri Do, MPH, MD	Medical Director, Asian and Pacific Islander Wellness Center	Asian American and Pacific Islander MSM
5	Geri Donenberg, PhD	Associate Dean of Research<br>School of Public Health, University of Illinois at Chicago	Design of HIV prevention interventions for youth
6	Lisa Hightow-Weidman, MPH, MD	Clinical Associate Professor, Division of Infectious Diseases University of North Carolina School of Medicine	eHealth interventions for Black MSM

7	Errol Fields MD, MPH, PhD	Assistant Professor of Pediatrics John Hopkins University	Black YMSM
8	Sebastian Linnemayr, PhD	Economist (Full); Associate Director, Research and Policy in International Development (RAPID), RAND Corporation	Behavioral economics of HIV interventions
9	John Lowe, RN, PhD, FAAN	John Wymer Distinguished Professor College of Nursing, Florida Atlantic University	Oklahoma Cherokee MSM; Designing culturally grounded intervention for AIAN youth
10	Harlan Pruden	Co-founder of the NorthEast Two-Spirit Society, Presidentia Advisory Council on HIV/AIDS	First Nations Cree; LGBQ-Two Spirit leader, scholar and activist

Through engagement with our scientist and community leader expert panel members we will identify language, images, formatting/visual cues, and examples in the current MyPEEPS curriculum that require cultural consideration for each racial/ethnic group. We will work with members to incorporate diverse views and reach censuses on common concerns, risk and protective factors, while providing a succinct mobile app respectful of cultural diversities. Dr. Schnall will facilitate the review of the MyPEEPS curriculum, assisted by Dr. Garofalo. The curriculum will be displayed on a screen, and each section read and discussed. Questions will probe for appropriateness, specific examples for a younger group and content to ensure that it is relevant to local conditions including: rural populations, American Indians, Pacific Islanders, African Americans, Latinos, and Asian Americans. As a multi-ethnic intervention particular feedback will be elicited by end users and experts with expertise targeting Asians, Pacific Islanders, American Indian and Alaskan Native MSM, as they were not represented in the MyPEEPS pilot. Dr. Pearson who has considerable expertise working with these populations of YMSM will take notes inserting changes directly into the curriculum viewed on the screen, supplemented by note-taking by study support staff and audio-recorded. This allows us to confirm meaning, make revisions as necessary, and reach consensus with the group. Dr. Garofalo will also monitor for cultural differentials, probe for further discussion on feasible ways forward and to ensure that it meets the current HIV prevention guidelines. As a final step, we will send out the final version of the MyPEEPS content to the expert panel to review after the meeting and return any comments or additional edits within 2 weeks.

The MyPEEPS Mobile system will be developed in English only. Despite the inclusion of a diverse population, recent census data showed that among 15 to 19 years old, 83% of those who spoke Spanish and 81% of those who spoke a language other than Spanish spoke English "very well,' and among 5-14 year olds, English "very well'. These numbers suggest that >80% of our target population who speak a language other than English are still able to speak English very well.

After the Expert panel meeting, revisions will be incorporated into the MyPEEPS curriculum and then we will conduct regional in-depth interviews with YMSM to obtain their feedback.

B.1.2. In-Depth Interviews with YMSM

Purpose 1: The goal of these in-depth interviews is to work with YMSM to design the user interface so that MyPEEPS Mobile will be usable for our intended end-users.
interface so that prototypes can be created at the end of the design sessions.

Recruitment: For this component of the study, we will recruit up to 20 YMSM from each of our sites (New York, Chicago, Birmingham, and Seattle). We will recruit YMSM through flyers (Appendix C) and information cards, and through referrals from local agencies, organizations, and other study participants. Sample: We will conduct individual in-depth interviews. Using purposive sampling, we anticipate that a total of 40 participants will be an adequate number of participants to reach saturation based on our earlier studies focusing on similar topics.[32] Procedures: Participants will receive $25 as a token of appreciation for their time during the 90 minute in-depth interview. Food appropriate for time of day will be served during the interview. As in our previous work development of technology for HIV prevention and management,[17,30,32,63] all interviews will be audio-recorded. The interviewer will take notes. If the participant is unable to visit the study team in person, interviews may be conducted via phone or Skype. In this instance, a verbal phone assent will be collected. We will first explain the

purpose of the project as well as the existing MyPEEPS intervention. We will ask structured questions related to areas of content that need further development or updating from the original MyPEEPS curriculum as well as functionality and dosing: 1) Over what time period would you complete a module? 2) How long do you want to spend using MyPEEPS Mobile each day, week? 3) At what time of day are you most likely to use MyPEEPS? 4) To inform recruitment for our trial, we will ask what websites and apps potential participants use. Following these questions, we will ask each group to sketch a user interface of the desired mobile app. Participants will be asked to describe the organization of the content and features and the desired "look" of the user interface they would want to see in an app. After participants share their ideas, we will ask probing questions to stimulate discussion about the content, features, and interface design. Participants will also be asked to identify platform requirements and navigational features.

Transcription: All design sessions recordings will be transcribed verbatim through a transcription company. Any identifiers will be deleted from the audio and removed from the transcript. Data Analysis: The study team will meet and review transcripts and notes. Drs. Schnall and Hidalgo[17,21,62-64] who both have experience in qualitative analysis will work with two research assistants to code the transcripts. Field notes and transcripts will be analyzed by the researchers using NVivo™ (QSR International, Victoria, Australia) software. Participants' statements will be captured using memoing and then sorted into the categories of interest. Open coding will be used to develop initial data categories. Some codes will be derived from the questions included in the interview and other codes will emerge from themes and patterns identified in the narratives. An initial set of codes will be independently generated by two coders. Codes will then be compared and synthesized to result in shared coding categories and sub-categories, all with definitions, inclusion and exclusion criteria, and examples. Coders will discuss discrepancies until they reach consensus. The study team will also develop a set of use cases based on the end-users plans for use of MyPEEPS. A use case is a list of steps, typically defining interactions between an end-user and a system to achieve a goal.[65] The study team will meet and review transcripts, notes, and drawings from the second focus group session and create a paper prototype of the system to give to our development team at Little Green Software (LGS). Scientific Rigor: The team will adhere to qualitative research processes to ensure the credibility, confirmability, dependability and transferability of the qualitative data from these analyses. To support the credibility of the data, we will conduct peer debriefing. We will also use "member checks," i.e., sharing of initial data interpretations with participants to ensure accurate interpretations. Triangulation of findings, along with reflexivity, will enhance the confirmability of the interpretations. The investigators will carefully record an audit trail and keep extensive field notes to facilitate transferability of study findings into other contexts.

B.1.3. Development of mobile delivery technology.

Following the in-depth interviews, we will develop mock- ups of a MyPEEPS Mobile with our partners at LGS. The mockup will have partial functionality of Garofalo and Hidalgo to develop the vision for the technical components of this intervention. A team of software engineers and project managers at LGS will perform the software development and the text messaging platform that will be required for this study. LGS is uniquely qualified to build the technical components of the MyPEEPS Mobile system. The Columbia University team will have a dashboard to monitor the delivery of the MyPEEPS Mobile intervention. MyPEEPS Mobile will be novel, innovative, scientifically sound, scalable, translatable and likely to have a strong public health impact, as detailed in the Innovation section.

B.1.4. Refinement of the MyPEEPS Mobile user interface.

Following the development of MyPEEPS Mobile, we will conduct Usability Evaluations.

Usability Testing. The goal of usability evaluation is to improve the design and increase the likelihood of technology acceptance. We will evaluate the user interface and system functions of the MyPEEPS Mobile System developed at LGS and assess whether they are consistent with the end-users' needs. We will conduct two types of usability assessments: A) Heuristic Evaluation and B) End-User Usability Testing.

B.1.4.a Heuristic Evaluation.

Sample: Five informaticians with training in human-computer interaction and who have published in the field of informatics will be recruited as usability experts. Nielsen recommends using three to five evaluators since one gains little additional information by using larger numbers.[66] We will recruit them through direct contact from the Informatics Departments at Columbia University and Weill Cornell Medical College, both of which have large cadres of informatics researchers. Procedures: The usability experts will be provided with a Beta version of the MyPEEPS Mobile Intervention. Similar to procedures that we have used in our prior work,[31,39,67,68] each usability expert will be asked to evaluate the system using the Heuristic Evaluation Checklist (Appendix B) and to think-aloud while performing the usability testing.[69] The process will be recorded using Morae software™ (Techsmith Corporation, Okemos, MI).[70] Participants will be asked to say aloud what they are thinking, seeing and trying to do while they are performing the tasks required for the scenarios. If a participant is silent for a long time, the researcher will remind them to think out loud. When a user finds errors or the researchers finds critical incidents that are characterized by comments, silence, or looks of puzzlement, the researcher will record the users' activities. Recording the users' interactions and vocalizations provides additional feedback that can highlight problems that would not be identified with static screen shots.[71] Instrument: Nielsen[72] proposed a list of ten recommended heuristics for a usable interface design. Bright et al.[73] developed a Heuristic Evaluation Checklist based on Nielsen's ten heuristics.[69] Each heuristic will be evaluated by one or more items and the overall severity of the identified heuristic violations will be rated. Data Analysis: The frequencies of usability issues will be calculated according to the heuristic principles adapted from Nielsen's checklist. Mean severity scores will be calculated for each heuristic principle. Evaluators' comments about usability problems on the evaluation form and the Morae recording will be grouped and content analyzed according to the usability factors of Nielsen's heuristics.[74] Based upon the findings of the heuristic evaluation, the user interface of the website and the messaging system will be refined by LGS.

B.1.4.b. End-User Usability Testing.

We will conduct usability testing with YMSM to identify violations of usability principles and any potential obstacle to their effective use of the MyPEEPS Mobile system. This is an iterative process that involves testing the system and then using the results to change it to better meet users' needs.
Sample: We will recruit 20 YMSM (13-18 years) in NYC, Chicago, and Birmingham, AL to participate in the formative evaluation of the prototype user interface screens. In a study examining benefits of increased sample sizes in usability testing, researchers found that the minimum percentage of problems identified rose from 82% up to 95% when the number of users was increased from 10 to 20.[75] Procedures: Participants will use MyPEEPS Mobile in our usability lab. All participants will be provided with scenarios and asked to complete tasks using MyPEEPS Mobile system on a laptop. While they are doing the tasks, their eye movements and laptop screen will be recorded using iMotions eye tracking software (i.e. Tobii X2-30). During each task, the start and stop times of tasks will be also measured. After completing the tasks, they will be asked to think aloud and verbalize their thoughts about the tasks they completed, through a replay of the screen recordings. Participant's reactions and verbal comments will be video and audio recorded. As part of the usability assessment, participants will complete electronic participants will be compensated $40 for their time.

Instrument: We will measure self-reported ease of use and usability with the Health Information Technology (IT) Usability Evaluation Scale (Health-ITUES).[76,77] This tool varies from most traditional measurement scales in that it is designed to support customization at the item level to match the specific task/expectation and health IT system while retaining standardization at the construct level. The Health-ITUES supports evaluation of three levels of task/expectation: user-system, user-system-task, and user-system-task environment. Schnall has published on the usefulness of the Health-ITUES for eye tracking recordings of user sessions, transcriptions, notes, and the user surveys and mean task performance time will be calculated. Dr. Schnall will search for

critical incidents which will be characterized by comments, silence, and repetitive actions. Schnall will review these incidents in detail using iMotions eye tracking software. The incidents will be identified and the users' written comments summarized. Content analysis, a technique for making replicative and valid inferences from data, will be performed by the research assistant under Schnall's supervision. The comments will be categorized according to the positive characteristics, negative characteristics, and recommendations made by the end-users. Results from the standardized surveys will be analyzed using SPSS (IBM, Armonk, NY) to calculate the descriptive statistics to complement the findings from the usability assessment. Using the findings from these activities, we will refine MyPEEPS for use in the pilot study (Aim 2) and the RCT (Aim 3).

B.2. Specific Aim 2: Pilot test MyPEEPS Mobile in a sample of 40 YMSM (10 at each study site).

Purpose: The goals of the pilot study are to: a) Gain direct feedback from participants about whether and to what degree the MyPEEPS Mobile system worked as intended; b) Assess the acceptability of MyPEEPS Mobile dosing and content across diverse YMSM; and c) Observe the flow of procedures, including assessment at baseline, retention efforts, data management, and follow-up assessments.

Study Design: The pilot study will be a 6 week pre-posttest design with 40 MSM across 4 sites.

Recruitment: We will recruit a convenience sample using mixed approaches including both active and passive recruitment methods described below. We will also use snowball sampling, a nonprobability sampling technique where existing study subjects recruit future subjects from among their friends, to identify additional participants until the desired number is recruited, as our study team has completed in their previous work (See Table 4). Each man approached will be given a project information card or flyer that provides contact info for the study site and basic information regarding the study. Study team members will complete the pre-screening form for active recruitment in outreach (via REDCap or paper form if no WiFi available) (Appendix C).

Participants may also complete the pre-screening form online via REDCap. Potential participants will be given information about the general nature of the study trial, the time involved (number of visits and approximate length of time: 1.5-2 hour survey completion at baseline and 6-week follow-up). We will be compensating participants for time and travel: $35 for baseline visit, and $45 for 6-week visit, + $25 per module completed ($25 x 3 modules = $75 max) = $155 total max compensation. The visit compensation is graduated and consistent with payments in prior studies.

B.2.1. Pilot Study Cohort:

We plan to recruit 10 participants from each site (Birmingham (AL), Chicago, NYC, Seattle) by posting flyers at our local partner sites (See letters of Support). We may also use online recruitment including posting on social media platforms such as Facebook, Instagram, and/or Grindr.

B.2.2. Screening*:*

Upon arrival at the enrollment visit and prior to written informed consent procedures (described below), all individuals will be screened for eligibility in a private location. This will confirm that the participant walking in is the same as the participant screened and scheduled when recruited, and to re-assure eligibility for participation in the study. They will provide verbal informed consent to participate in a brief screening interview also as described above. Individuals who screen eligible will then complete written informed consent procedures. Those who do not screen eligible will be informed and thanked for their time.

B.2.3. Written Informed Consent Process for Study Participation.

We will use written informed consent/assent procedures for enrolling YMSM into the pilot study. At the first visit, participants will be handed an informed consent form and the study staff will read the form to them. To ensure study procedures. The informed consent form provides details of the study procedures, risks, benefits, site contact information, and the nature of confidentiality and voluntary participation. The consent process also covers information on the trial timeline, task

involved, and compensation for time as outlined in the Screening section above. Before a participant signs the informed consent form, staff will answer any questions. Participants will be given a copy of the informed consent form for their records.

B.2.4. Secure Web-based Baseline Assessment.

After providing written consent, the participant will complete the baseline assessment on a computer or tablet in a private location. The baseline assessment consists of a ~1.5-hour web-based survey with demographic and outcome measures listed in Table 3 (Appendix B). These measures have all been previously used and tested in studies with adolescents and YMSM and were tested in our the MyPEEPS pilot study.[5] We are using a web-based survey software package (Qualtrics). Benefits to Qualtrics[78] include data being captured directly in electronic format and interactive data capture checks, beneficial in a large multi-site trial as it will allow for secure and consistent data capture across sites. Qualtrics is a web-based system that provides an intuitive interface, audit trails, and automated export and is free through Columbia University. Staff at each site will sign-in to the secure web-based data collection survey tool, provide the participant with preliminary instruction on its use, and will remain easily accessible during survey completion to address any technical problems or to answer questions participants may have. Staff may periodically check in with the participant to inquire about any difficulties that may arise in completing the survey, breaks and snacks will also be provided. However, to ensure privacy, the staff person will not directly observe the full process of completing the survey unless requested by the participant.

B.2.5. Intervention Delivery.

Participants will have 6 weeks to complete the MyPEEPS Mobile modules. The research assistants at each site will monitor the progress of their participants. Study staff will have access to a dashboard which will only list the study participants at their site and monitor module completion. In addition, participants will receive weekly text messages and/or e-mails (participants will select) to remind them to complete the MyPEEPS module. If 2 weeks have passed and a participant has not completed any modules then study staff will call participants to remind them to complete their modules. After consenting to participate in the study and completing the baseline surveys, the research assistant at each site will download the MyPEEPS Mobile app to the participants' smartphone and give the participant a username and password. Each module is estimated to take about 60-90 minutes. Study participants can log in at their convenience and will not be able to access the next module until the previous module has been completed. Participants cannot do more than 2 modules/week and will receive a token of appreciation for completing each module. Each participant will have a login and password and to prevent his parents from being able to login to the application and view its content. The benefit of reducing sexual risk behaviors in our study population outweighs the potential harm of parents overseeing their child's use of the MyPEEPS application. After logging into the MyPEEPS Mobile, participants will be guided through the curriculum by Phillip aka "P". MyPEEPS avatars represent "regular guys" like the study participants who will walk the participants through the curriculum. At the start of each module, a MyPEEPS character will present his Peeps Problems. In the pilot, there were 4 MyPEEPS characters (Figure 4) who were designed based upon the social realities of YMSM based on our formative work. They include: Art (Artemio) has a girlfriend who doesn't know that when Art says he's with "the guys", he's really "with" guys. Art uses the pull out method when having sex with his girlfriend and only uses condoms with guys, since he believes that "only gay people get AIDS." Philip (a.k.a. "P") recently came out as Bi to his close friends, and is looking to meet guys to hang out with. Philip isn't out to his family and most of his friends because he knows they will not accept him. Nico is completely out and proud, but sometimes struggles with feeling like he's juggling between two races. He loves dude/bros (White, preppy, frat types). Tommy lives with an older guy who helped him get on his feet when he first came to the city. Though Tommy is healthy and lives in a great

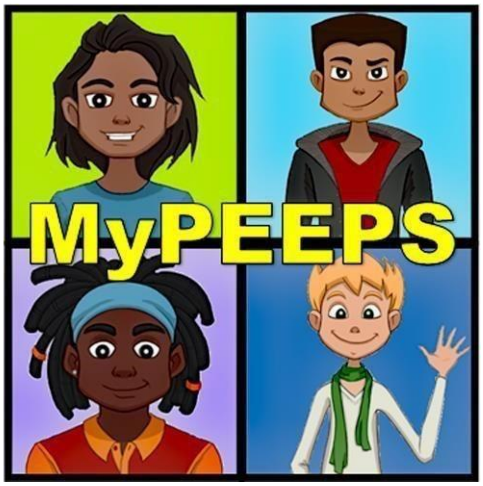

neighborhood, he's not happy. His boyfriend's jealousy keeps Tommy from meeting other guys his age. A scenario from the life of one of the Peeps lives will be presented at the beginning of each module. A sample scenarios is: A couple that comes into my restaurant pretty often asked me if I wanted to have a three-way with them. I would have sex with her AND him, but I'm not sure how I feel about doing it at the same time. Am I missing the opportunity of a lifetime? Should I do it? As part of our formative work in this application, we will add additional characters from other racial/ethnic minority groups. MyPEEPS Mobile will have a menu of the 3 modules that will need to be completed in consecutive order. We estimate that each module will take 60-90 minutes to complete. Participants do not need to complete a module in a single sitting.

B.2.6.Secure Web-based Follow-Up Assessment.

After 6 weeks, the site staff will meet with the participants to complete a follow-up assessment which will include the primary and secondary outcome assessments that we plan to use in the full trial (Appendix B). To complement the quantitative assessment, we will conduct in- person debriefing interviews with participants following the assessment. Prior to the debriefing interview, participants will be able to review the MyPEEPS Mobile modules on their phones or a tablet at the site. During the interview, staff will review each module with the participant and ask followup questions pertaining to relevance, self-efficacy, comprehension, and technical difficulties in understanding the topic areas. The interviews will be recorded and transcribed verbatim through a transcription company. Any identifiers will be deleted from the audio and removed from the transcript. The goal is to collect critiques of the material, content, delivery methods, and to identify subject matter that should be included to enhance relevance and efficacy. We will also ask participants to make suggestions for improving MyPEEPS Mobile and to note which features they liked and disliked. Lessons learned from the pilot study will include if there are changes needed to how frequently we send text messages, changes to our outcome measurement tools, and training on how to use MyPEEPS Mobile and best practices for recruitment and retention. The procedures for the full trial will be modified as necessary based on the pilot findings.

B.2.7. Young Adult Pilot: Pilot Test MyPEEPS with 19-25 year old MSM (n=10 at each of 2 sites: NYC & Chicago)

We will pilot the MyPEEPS Mobile app with young adults ages 19-25.

Sample: We will recruit 20 YMSM (19-25) in NYC (n=10) and Chicago (n=10) to participate in the pilot testing of the MyPEEPS App. Participants will be recruited using flyers and online posts. Participants will be screened to determine eligibility.

Procedures: Participants will complete all 21 activities within the MyPEEPS app in-person at the study visit. Participants will also complete an electronic survey via Qualtrics following the testing of the app. Participants will also answer interview questions about the app, which will be audio recorded. The visit will take approximately 3-4 hours and participants will be compensated $125 for their time. The audio recording will be transcribed, and any identifiable information removed.

B.2.8. Multi-site Focus Groups: Conduct Eight Sessions with Female Assigned at Birth (FAB) Trans Masculine & Gender Non-Conforming Youth, 13-18 Years Old, to Assess the Relevance and Adaptability of MyPEEPS Mobile

Objective: We will conduct multi-site focus groups with female assigned at birth (FAB) adolescents who identify as female-to-male (FTM) and along the trans masculine spectrum or as FAB gender nonconforming, ages 13-18, in order to assess the relevance of the MyPEEPS Mobile app and better understand how the app would need to be adapted for this population.

Sample: We will conduct eight multi-site focus group sessions with up to 60 trans masculine and gender nonconforming youth, approximately 6-12 participants per group. We will conduct 1 focus group in Birmingham, 2 in Chicago, 4 in NYC, and 1 in Seattle. Participants will be drawn from the group of youth who screened through our MyPEEPS Mobile RCT, but were ineligible because they

indicated female sex assigned at birth. We will also flyer to recruit the rest of the study sample if we cannot enroll enough individuals using the MyPEEPS Mobile RCT screener. Participants will be screened online through REDCap or over the phone using a pre-enrollment screening form by the research staff to determine birth; 1) identifying as female to male (FTM), transgender man/male, trans masculine, or FAB gender nonconforming/non-binary; 2) understand and read English; 3) live in the US; 4) have smartphone; 5) self report sexual interest in non transgender (cisgender) men and has either kissed non-transgender (cisgender) man or thinks they will have having sexual activity with a non-transgender (cisgender) man in the next year; 6) and self-report HIV negative or unknown status. Participant *exclusion* criteria includes: 1) younger than 13 or older than 18 years of age; 2) male sex assigned at birth; 3) identifying as (cisgender) to a smartphone or tablet; 4) are living with HIV; 5) investigators determine that participation may be detrimental to the participant or to the study (e.g., severe cognitive deficit).

Procedures: Following completion of the electronic informed assent/consent process, participants will be asked to: 1) complete the 21 activities within the MyPEEPS Mobile app in-person using their phones or tablets provided at the site before the survey 2) complete an electronic survey via Qualtrics after completing the MyPEEPS Mobile app but before the focus group session; and at the end, 3) partake in a focus group session which will be 1-1.5 hrs in length and audio recorded. The PIs, who have conducted focus groups for a number of studies in the past, and their research team members, will convene groups with study participants and act as facilitators.[30,31,63,67] The focus group guide will include questions pertaining to the relevance, self-efficacy, comprehension, and technical difficulties in understanding the topic areas of MyPEEPS Mobile app. Furthermore, the guide will be informed by the feedback and commentary from members of our investigative team who have extensive clinical and research experience working with transgender youth. Some of the sample focus group questions include: 1) 'Thinking back about the information you learned from the MyPEEPS app, how would you apply this information/lessons/activities in your own life?'; 2) 'How do the MyPEEPS activities reflect your beliefs, norms, values?'; 'In what ways do you think this app would be relevant or irrelevant for young trans and non-binary adolescents ages 13-18?'; 4) 'How would you change the app to make it relevant to young During the focus groups, participants will be able to refer their notes taken while they completed MyPEEPS Mobile activities. We will also ask participants to make suggestions for improving MyPEEPS Mobile and to note which features they liked and disliked. The goal is to collect critiques of the material, content, delivery methods, and to identify subject matter that should be included to enhance relevance and adaptability. In length, all research activities related to the focus groups are expected to take approximately 4-5 hours. Participants will be compensated $150 as a token of appreciation for their time.

The team will adhere to qualitative processes to ensure the credibility, conformability, and dependability, and transferability of the qualitative data from theses analyses. To support the credibility of the data, we will conduct peer debriefing and triangulate findings across multiple data sources (surveys, focus group data). In addition, we will use "member checks," i.e., sharing of initial data interpretations with participants, to ensure accurate interpretations. Triangulation of findings, along with reflexivity, will enhance the confirmability of the interpretations. The investigators will carefully record an audit trail and keep extensive field notes to facilitate transferability of study findings into other contexts (Guba, 1981). Each focus group audio recording will transcribed and any identifiable information removed. Transcripts will be analyzed independently for content by research team members in these methods.

Data Analysis: Field notes and transcripts will be analyzed by the researchers. Participants' statements will be captured using memoing and then sorted into descriptive thematic categories. These activities will result in a greater understanding of relevance, adaptability, and development of MyPEEPS Mobile with FAB trans masculine and gender non-conforming adolescents, 13-18 years

of age.

B.2.9. Pilot test a MyPEEPS Art Contest to Involve Native and Indigenous Youth in the Design of MyPEEPS Mobile recruitment material.

Objective: The MyPEEPS Art Contest will use a community participatory approach to involve American Indian and Alaskan Native youth in the MyPEEPS Mobile project recruitment material. The contest will be used to select four pieces of original art that will become the basis of additional recruitment materials for the project.

Procedures:

- We will use existing recruitment channels in order to aid in advertisement of the contest: (a) Tribal health organizations; (b) Indigenous community organizations; (c) Community members who support Indigenous youth; (d) Other youth serving community organizations; (e) Social media platforms (e.g. Facebook, Instagram, etc.) The MyPEEPS Art Contest flyer (attached to the protocol) will be used for all advertisement of the contest. People and organizations contacted by the MyPEEPS staff will be asked to display the flyer or share the flyer's information within their own networks. The flyer will be adapted to for digital display and distribution in social media platforms. Promotion of the contest will last three months, during which time any youth artist who meets the eligibility criteria may submit and electronic version of their artwork through the REDCap website hosted at the University of Washington. Once the three-month period is complete, there will be a one-month voting period prior to the selection of the winning pieces.
- For the art submission process, we plan to use the University of Washington's REDCap system to capture contestant entries. In REDCap, contestants will have the ability to respond to the eligibility questions, enter their contact information and submit an electronic copy (.PDF, .PNG, .JPEG, or .GIF files) of their artwork (REDCap questions attached to the protocol). It was determined that an electronic art submission during the three-month contest period is the simplest way to ensure there are low-to-no barriers in youth accessing the contest. Electronic versions of original drawings, paintings, mixed media, and digital art will be accepted.
- Art Contest Eligibility: To be eligible, contestants must meet the following criteria: (a) Be between the ages of 13-18; (b) Self-identify as American Indian, Alaska Native, Native Hawaiian or from another Indigenous community
- Selection of Art Contest winners includes a two-phased approach to selecting four winning art pieces: (1) *Phase One – Open Voting:* use a popular-voting method to select the top ten art pieces from all those submitted at the closure of the advertising/submission period. Once the submission period is over, we will upload the submitted art pieces to REDCap to track voting and rankings of the art pieces. The link will be shared with the same contacts and communication channels used to advertise the art contest and will be available for one month for voting. At the end of the voting period, the top ten selected pieces will be brought to a MyPEEPS judging panel who will determine the final four winners;

  (2) *Phase Two – Panel Judging (Selection of the four winners)* The MyPEEPS judging panel will consist of members of MyPEEPS project staff, coordinators & investigators, as appropriate. The MyPEEPS judging panel will select and rank the top four submissions. The judging criteria is as follows: (a) The art work fits with the overall contest theme: Native sexual minority youth empowerment; (b) the appropriateness of the art work: no overt sexual images are used and content reflects the contest participant target age range (13-18 years); (c) the art work incorporations key aspects of the MyPEEPS study which are as follows: male identified health, youth, identity, culture and technology. The top four winning artists will be notified via email or phone that their art work has been selected and will be asked to complete an ownership agreement form (see Media Release Form attached to the protocol) that will grant ownership to the MyPEEPS Mobile Project / Columbia University, University of Washington and its assignees and licensees to use their submitted art work

and name. Once the ownership agreement form is signed, we will publicly announce the four winners.

- Only contestants whose art is selected as 1st, 2nd, 3rd, or 4th place will be provided a monetary compensation. No additional compensation will be provided to contestants who do not place. The dollar amounts for winning are per piece of art, not per person. If a single piece of art was submitted by more than one person (a group piece), only one prize amount will be rewarded. The identified prize amounts are as follows: (a) first place - $500; (b) second place: $250; (c) third & fourth place: $125/each. Once selected, the MyPEEPS staff will provide each winner with the appropriate monetary compensation and pay to ship the original pieces from the contestant to the University of Washington.

C. Specific Aim 3: Conduct a randomized 2-group multi-site RCT to evaluate the efficacy of MyPEEPS Mobile intervention to reduce HIV risk behaviors in 900 YMSM at 4 study sites.

C.1. Overview of the Study Design.

The purpose of the **multi-site RCT** is to test the efficacy of MyPEEPS Mobile as compared to delayed intervention arm. In comparison to a traditional 2-arm RCT, our study design includes an intervention and delayed intervention group with multiple post- tests in the case of the intervention group and multiple pre-tests in the delayed intervention group. Using the proposed design, we have larger statistical power to detect a difference in intervention effects and we can compare between

Figure 5. Study design timeline, assessment points and intervention delivery time points

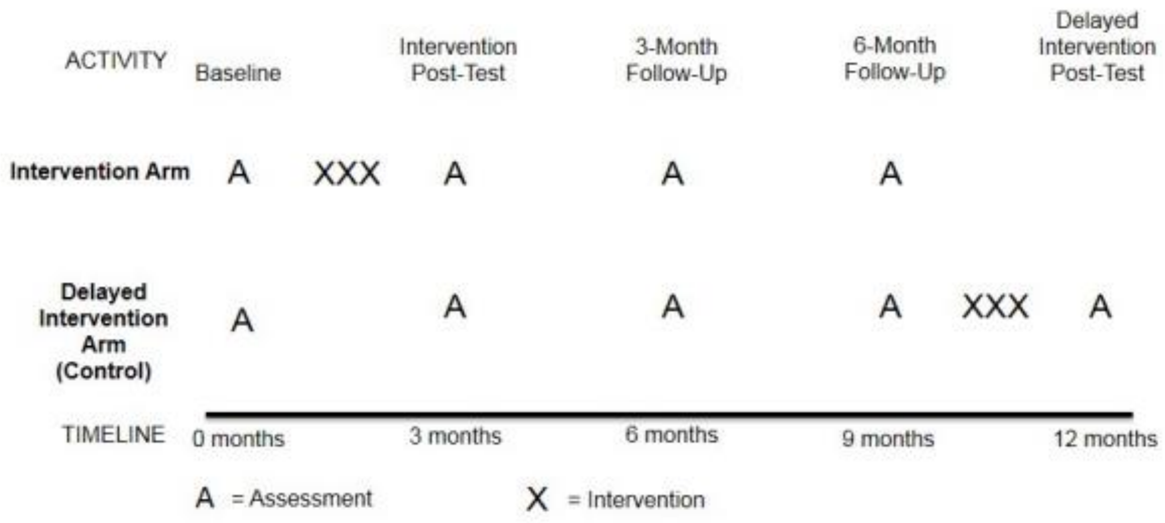

the intervention, since we have additional data points we have additional power to detect significant differences between both the intervention and delayed intervention group as well as a pre-posttest difference within groups. The multiple data points that we will collect from our delayed intervention group before exposure to the intervention will allow us to control for selection-history. This is particularly relevant for in the case of a mobile intervention since the mobile app marketplace is rapidly evolving and changing and so a new HIV prevention app may enter and/or leave the marketplace during our study. We will be able to control for this historical bias with our study design. While we have systems in place during our randomization to control for assignment bias, this threat to validity can still exist. Since both groups will receive the intervention, there is a mitigated risk for assignment bias.[90] Finally, we will be able to assess the 3 and 6 month effect of the intervention. The intervention outcomes are based on the content of the MyPEEPS Mobile intervention and match those from the MyPEEPS pilot. The study design, assessments, and intervention delivery time points are delineated

in Figure 5. The trial includes screening, baseline assessment, a 3 month MyPEEPS Mobile intervention and 3 and 6-month follow-up assessments. Recruitment for the study trial will last approximately 2.5 years. Participants will be able to attend the baseline visit in person or can set up a video chat (eg. skype, zoom, facetime call) through which we will verify participants identity and then we will send them a link to the e-consent form, baseline survey and login for the app.

C.2.Sampling approach to successfully enroll YMSM participants in this national project.
We will recruit 900 participants. We will no longer be limiting our participants to these study sites and have expanded to include study participants from across the US. Using active and passive recruitment methods, the on-site project coordinator at each site will oversee and participate in recruitment efforts. The study staff hired through this project, on-site coordinator and the peer-to-peer networking efforts at each site will be used to recruit study participants. Each of our sites have previously participated in recruitment efforts for projects similar to the one proposed here and will be able to successfully recruit the study sample for this project. Our study participants will all have face-to-face contact with our study staff as a means to verify participants' identity. In order to accomplish this using an online sample, we will verify participants' identity using video conferencing (i.e. Zoom).
Note: As per the RFA, we will ensure that we have an adequate number of participants in each age group (e.g., 13 year olds) to conduct valid sub-group analyses. Based on our power analysis, we will recruit at least 70 participants in each age.

C.3. Procedures for Maximizing Research Integrity.
To Maintain Blind Assessment, we will collect data through electronic surveys on tablet computers. Interviewers will remain in the room to ensure comprehension. Electronic survey completion offers several advantages: (1) It yields more accurate responses than face-to-face interviews about sensitive topics. (2) Participant can complete measures at their own pace. (3) Questions are answered privately increasing anonymity. (4) The interview structure permits skipping follow-up questions when the stem is answered negatively. (5) Computer delivery ensures uniform administration. Procedures for Maximizing Retention. We will use multiple strategies to reduce attrition.[79,80] At baseline and follow up interviews, we will update contact information and encourage ongoing communication. We will offer multiple reminders via text, email or calls, based on participants' preference. To ensure follow-up assessments can be conducted for those who move, we will provide access to the assessment via link into a secure server, where data is encrypted at entry and no identifiers are collected.

C.4. Description of study population.
The study population will consist of a diverse group of YMSM who live across the US. Each site will recruit an ethnically and racially diverse sample to include Blacks/African Americans, Hispanics/Latinos, American Indians/Alaska Natives, Asian Americans, Native Hawaiians and Other Pacific Islanders. We will also recruit "special populations" as described in our letters of support including: homeless youth and substance users. Race/ethnicity as well as these other special study populations (homeless or unstably housed, foster care, substance use) will be monitored throughout the study and recruitment will be adjusted and targeted with the intent of recruiting enough participants from each designated US health disparity population in sufficient numbers to conduct valid subgroup analyses.

C.5.Sample Size and Power Calculation.
We estimated the statistical power for the primary outcome of: number of condomless anal sex acts with male partners during the past 3 months (count outcome) based on the data from the MyPEEPS pilot study (R34MH079707). We estimated the statistical power for two scenarios: (1) to examine overall effect with total subjects; and (2) to conduct stratified analysis to examine the effects in some subgroups (such as by sex and/or for some racial/ethnicity subgroups). The following assumptions are used for the power estimation: (1) an 80% retention rate, at a single follow-up assessment for each

study arm; (2) a conservative and high intra-cluster correlation (ICC) of 0.8, (3) mean number of unprotected anal sex acts with male partner partners with whom a condom was not used during the past 3 months at the baseline is 1.2,[5] (4) all power estimations are based on $\alpha=0.05$ and 2-sided tests.

Findings from our MyPEEPS pilot study indicate that the post-intervention number of condomless anal sex acts during the past 6 weeks decreased by 63%, or a relative risk (RR) of 0.37.[5] However, the large effect was not statistically significant. Because the estimated effect size of the intervention was unreliable, instead, we use RR=0.73, one standard error over the estimated RR of 0.37, as the effect size for the examination of overall effect of intervention for total sample. This would provide a conservative estimation of minimum sample size need. For the subgroup analyses, we use the effect size of RR=0.37. To examine overall effect with total subjects, we expect that the post-intervention number of unprotected anal sex acts 27% (i.e., RR=0.73). We will have 97% power to detect such difference for the total sample size of 700. To test absolute relative difference (the equivalent margin) of <10% for the outcome with n=700. Secondly, for the stratified analyses, we will have 92% power to detect a relative risk of 0.37 in subgroups with sample size of 70. As per the RFA, we will have adequately powered comparisons that can be done separately for racial/ethnic groups, age (by year), geographic region (Northeast, South, Midwest and West), and socioeconomic status. In order to achieve this, each subgroup will need to have about 10% of the total subjects. For example, we anticipate recruiting 70 American Indians across all sites so that we will have enough power to detect a significant effect of the MyPEEPS Mobile intervention (See Planned Enrollment Table). To test whether the intervention is effective during the follow-up period, we will have 61% power to detect an absolute relative difference (the equivalent margin) of <10% or 95% power to detect an absolute relative difference of <15% for the outcome with n=70. However, we will increase target enrollment to n=900 to provide a better possibility of reaching the above indicated sample sizes for each subgroup.

C.6.Sampling Approach.

Our multi-site RCT sample will be a convenience sample through active recruitment at other venues including CBOs, schools and through social networking sites. We considered other sampling options such as “online only” that might have for instance allowed for the recruitment of a “national” sample - but in our experience this type of recruitment would have proven very challenging particularly in reaching racial ethnic minority youth in sufficient numbers. As such we believed a multi-site RCT grounded in 4 geographically distinct center was the best approach to take for testing the proposed mobile intervention. We will enhance our recruitment method to include recruitment through social networking sites utilizing a “national” sample as social networking sites extend to individuals outside the 4 main city-centers and we are therefore able to recruit an ethnically and racially diverse sample of YMSM.

C.7.Recruitment Approach.

We have successfully used these recruitment strategies in the past with YMSM in the cities selected for this project. Dr. Hirshfield will provide guidance/input on the development of the online recruitment procedures as well as implement the day-to-day interaction with our online partners during the recruitment period. In addition, PHS staff will facilitate communication between the study team and online vendors/partners based in the four recruitment cities.

C.7.a. Recruitment Venues.

Planned offline and online recruitment venues include the following: Community Outreach: Peer outreach staff will directly approach and recruit potential participants from local beaches, parks, gyms, coffee houses, clubs, house balls, and social establishments that cater to or are frequented by YMSM in the four cities. In community venues that cater to YMSM, trained, staff will approach potential participants, describe the study, and obtain oral consent or assent to screen for eligibility.

Community Presentations: Across sites, study staff will attend and/or make presentations about the

study at selected activities and social events sponsored by our community partners. When making presentations at selected events, the recruiter making the presentation will provide a brief background on the study, and invite all potential participants who are interested to speak to one of the study recruiters. With permission from local high schools, we will conduct community presentations and provide study flyers; thus far, we have letters from Broome Street Academy and Peer Health Exchange. We will also seek approval to present at LGBT events sponsored by programs such as Planned Parenthood.

Department of Health Clinics, Medical Clinics, and FQHCs: The study sites have strong ties to the public health community and will be able to advertise for the study in various clinics that serve the target population. Specifically, Columbia University and PHS will be able to advertise via flyers for the NYC study site at Columbia University Medical Center, as well as NYC DOHMH STD and HIV clinics (please see Letters of Support), and APICHA which serves racially/ethnically diverse MSM with an emphasis on Asian and Pacific Islanders (See Letter of Support). Chicago is equally well-connected to health clinics and youth resources such as the Peer Health Exchange (e.g. Chicago public schools), the Broadway Youth Center of Howard Brown Health, Illinois Safe Schools Alliance, Illinois Caucus for Adolescent Health, TPAN, BYC, Northwestern on Belmont, U of C Medical, South Side Health Center, and VIDA/SIDA. The Alabama site will recruit from their main CBO in Birmingham, Birmingham AIDS Outreach, through Advocates for Youth Birmingham. Seattle has strong relationships with Harborview public clinic and STI/HIV clinic, and Gay City community LGBTQ center.

Newspaper Advertisements: We will advertise the study through the placement of ads in a variety of free newspapers widely distributed throughout cities across the four study sites.

Craigslist Advertisements: We will post free daily ads on Craigslist

Social and Sexual Networking Web sites and apps: We will recruit participants online through various websites and apps including but not limited to Instagram and Snapchat which are frequented by youth. We will now include ads that will target the entire US and not limited to our 4 local study sites. We will target and recruit study participants through Facebook, which allows targeting of individuals down to age 13. For Facebook, we will use psychographic targeting (in-depth, publicly available consumer data such as interests, city) to advertise only to potentially eligible individuals across the US.

Blogs and YouTube: We will approach bloggers to advertise for the study on sites like: ShadeRoom.com or LoveBScott, and will buy ad space on YouTube for the channels with 'famous YouTubers' that resonate with our target populations.

Media Sites: The study will set up an Instagram profile to promote the study and we will also advertise through outlets like Tumbler, Periscope, and Snapchat.

CBOs/ASOs NYC Email Blast: PHS's Contracting and Management Services division has served as the "Master Contractor" for NYC Department of Health and Mental Hygiene (DOHMH)-funded Ryan White Part A programs. On behalf of the NYC DOHMH, PHS currently administers $105 million, representing >150 CBOs and ASOs, hospitals, clinics, and mental health clinics. In past studies, PHS has contacted the NYC CBOs and ASOs (via a confidential email list) that receive Ryan White HIV prevention and treatment funding to freely advertise for NYC studies using study-approved flyers and brochures, and proposes to do so for this study.

Dedicated Study Website: This site will present basic content about the study and contain a secure link (via REDCap) for participants to indicate their interest in participation. Participants who leave

their contact information via the website will be contacted by study staff to be screened for eligibility. Eligibility screening will only be done over the phone, online, or in person, **not** via email or text message.

Online Study promotion via Social Networking Sites: The study will establish and monitor social networking accounts (e.g., Facebook, Instagram, SnapChat) that will run during the study recruitment period and will promote the study among users who are 13-18.99 years old (based on social networking profile information). Study staff will post daily mass media content relevant to YMSM as well as intermittent study promotion content, and create paid advertisement campaigns promoting the social networking accounts. Study-specific promotional content will consist of IRB-approved language. All user private messages or comments related to study participation will be addressed by the study staff through the use of IRB-approved scripts that provide study screening instructions. No pornographic content or material suitable for adults aged 18 and over will be posted.

C.7.b. Potential Problems/Alternative Solutions Regarding Recruitment:
Consistent with the multiprong recruitment approach designed to reduce recruitment bias,[81] we will employ the aforementioned recruitment methods. To ward off potential recruitment problems, our team will carefully monitor each approach to prevent recruitment problems. This will involve weekly review of recruitment data with recruitment staff to assess efforts. In this way, recruitment will be a dynamic process and will reach the proposed diverse group of YMSM.

C.8. Online Study Procedures
We plan to enroll 900 13-18 year old male youth across the U.S.. We will recruit potential participants using both online social networking and sexual networking websites (e.g., Facebook, Grindr, BlackGayChat, Scruff, and new sites identified by pilot participants in Aim 2). Social networking and sexual networking sites are accessible to individuals across the US and do not require individuals to live in a particular city to access the information within. We will recruit through these networks using an open platform which will allow us to reach potential participants from all 50 states and not limit us to a particular city/state. Utilizing these open access platforms will allow us to expand the range of participants we can reach. Potential participants who click on an online survey banner advertisement will automatically be linked to the study landing page on REDCap that contains a consent form. Potential participants will be required to read the informed consent document before indicating whether or not they consent; this will be encouraged by requiring participants to scroll through the entire consent before they can choose to consent to the study. During the informed consent process, the minimal risks, the anonymous and voluntary nature of participation, and the fact that participants can stop their participation at any time are described. Clicking the consent button will indicate that the participant has read the consent form and agrees to participate. A button to allow participants to print the consent form for their records will be located at the end of the consent form document. We will provide our email and phone number contacts on the consent form in case participants have further questions about the study. Potential participants can exit the website at any time by closing their browser. Consent or lack thereof will be documented in the electronic database by the stored variable indicating consent or lack of consent.

Although Federal regulation requires that researchers obtain written informed consent for research on human subjects, under 45 CFR 46.117(c) written consent can be waived for research that involves minimal risk to participants and involves no procedures for which written consent is normally required outside of the research context. This research meets that criterion. We propose an alternative approach where participants click a button, signifying that they have read the informed consent page and agree to participate in the study. An advantage of internet-based studies is that the consent form is available for the subject to review and/or print at any time. This strategy complies with the requirement of 46.117(c) that participants are given a written statement describing the research and

risks. We have used these procedures without a known adverse event in 6 previous Internet studies, enrolling thousands of participants online.

Once online consent is provided, potential participants will complete a brief online screener survey (provided in English) to determine study eligibility. Potential participants will have the option to complete the online survey on their computer, Smartphone, or tablet. If ineligible, the individual will be automatically routed to a webpage that thanks them for their time and states that they are not eligible at this time. The webpage will contain links to national and local prevention resources, such as HIVtest.org, CDC.gov, and http://www.teenhealthandwellness.com/static/hotlines.

C.8.1. Eligibility screening.

Potential participants will either call, text or email the project via the project phone number or email address; or, if contact information is gathered at the recruitment venue, be called, texted or emailed by project staff. Screening will be conducted over the phone, online, or in person for walk-ins; screening will **not** be conducted by email. Each potential participant will be screened for eligibility using the full screening instrument. If a parent is present at the time of potential study recruitment or enrollment, such activities would only take place in a private location away from their parents. At the same time, the risk of negative parental involvement is mitigated if a parent is at the health visit with their child at a community health center such as Callen Lorde Community Health Center, as they are healthcare and related services providers targeted to lesbian, gay, bisexual, and transgender communities. If a parent is accompanying a child to a visit at a community health center with this focus, it is likely that the parents are already aware of their child’s sexual health practices.

The following verbal script is a guide to describe the screening process and privacy issues, as outlined in Appendix C: "Hi. I work with [e.g. Callen-Lorde Community Health Center] on a research study called MyPEEPS Mobile. Have I reached <insert name>? The study will involve (number of appointments questions. It will only take a minute or two. Some of the questions are personal. Your involvement is voluntary, your responses will be kept private, and you can refuse to answer a question or stop at any time. Information we gather from you will not identify you individually. Are you interested?” The full screening will only take a few minutes (Appendix C). If a potential participant is screened and found eligible and willing to participate then he will voluntarily provide written informed consent. Contact information will be destroyed if youth decline study participation; however, a count of ineligibles and declines (active and passive) will be maintained. Secured information about those who participated in the pilot phase will be referenced during trial enrollment in order to verify that they indeed did not participate in the pilot. If it is determined during trial enrollment that a young person participated in the pilot, they will be deemed ineligible to be in the trial and cordially told so.

C.8.1.a. Secondary Screening to Confirm Eligibility

Participant eligibility will be confirmed in person prior to obtaining informed assent/consent. For participants who are enrolled online, we will also use a form of video conferencing (eg. Zoom, etc) so that participants can show an ID and verify their identity.

C.8.2. Informed assent/consent.

Upon arrival at the enrollment visit we will collect written informed consent (18 year olds) (assent for 13-17 year olds) for enrolling men into the study trial (See Protection of Human Subjects and Inclusion of Children). The informed consent form provides details of the study procedures, risks, benefits, site contact information, and the nature of confidentiality and voluntary participation. The consent process also covers information on the trial and compensation for time. A baseline visit will be conducted involving a behavioral assessment (see questionnaires in Appendix A). The baseline assessment is expected to last approximately 1.5-2 hours and participants will be given $25 as a token of appreciation for their time. YMSM will then be randomized to the intervention or delayed intervention

arm.

C.8.3. Randomization to intervention and delayed intervention study arms.

Using the randomization approach described below, there will be random or minimally biased assignment of subjects to study arms. to We will use block randomization for this study,[82] stratified by site and each category of study participants with have randomly permutated blocks to reduce opportunities for selection bias we will use a variable permuted randomization block design where the block size itself is randomly selected (i.e., blocks of four to eight). The advantage of the permuted block design is that treatment assignment is predetermined before the trial begins and then assignment remains static throughout the course of the trial.[83] Blinding and random assignment will be maintained through continuous supervision by key members of the research team. All staff, and participants will be kept blinded to outcome measurements during data collection.[84] This technique will maintain complete randomness of the assignment of a subject to a particular group. Participants will be randomized based on the use of computer-generated random numbers at baseline.

The randomization database will be stored on a password protected computer at Columbia University and will only be accessible to Dr. Schnall and the project manager at Columbia University to avoid the possibility of the study sites subverting randomization as has been noted in previous studies.[85] Following completion of the informed consent and baseline assessment, participants will be randomly assigned to one of the two trial arms using sequentially numbered, opaque, sealed envelopes containing the intervention assignment, which the staff member opens at the moment of randomization.[83] This will minimize the biased assignment of study subjects which meets the PRS Efficacy Criteria for Best Evidence Risk Reduction Individual Level -Tier 1.[86] This will ensure against accidental bias and produce comparable groups in all respects except the intervention each group received.[87] If the study participant is randomized to the intervention arm, they will have access to the MyPEEPS Mobile for the next three months. Participants randomized to the delayed intervention arm will not have access to MyPEEPS Mobile and will be asked to complete their surveys every 3 months and will be provided access to the app at 9 months.

Participants in both groups will complete a baseline assessment (Appendix B) at time 0. Through our system, we will be able to monitor when participants login to MyPEEPS Mobile, time spent on each module and whether they are actively using the intervention or just logged in.

For participants enrolled online, once they are consented, we will then email them the baseline survey through Qualtrics and send them a login in to the app if they are in the intervention group. If they have not logged into the app within 24 hours after enrollment, we will call them to follow up.

C.9. Overview of data collection time points.

We will conduct simultaneous assessments for both intervention arms at three-month intervals starting with a common baseline through six-month postintervention follow-up (Figure 5). We will also collect an immediate post-intervention assessment for Best Evidence Risk Reduction Individual Level -Tier 1.[86]

Follow-up visits will be scheduled within a target window of 2 weeks before to 2 weeks after based on participant availability and site capacity; visits may be scheduled approximately one week before or up to about four weeks after the target follow-up date. Timing of follow-up visits is scheduled to allow site flexibility while still scheduling follow-up visits within a reasonable time span. If an individual misses one of the follow-up visits (e.g., 6 month visit for intervention group), then we will still attempt to collect their 9 month visit data. Every effort will be made by staff to ensure that participants are assessed in follow-up visits within, first, the target window, and second, the acceptable window. After consultation with the Co-I, the PI will decide how best to handle the situation for the individual participant and point in time that he contacted. For example, if a

participant was out of the country during the 3-month window and eventually calls the site at the 5-month point, the on-site study coordinator will consult with Drs. Schnall or Garofalo.

We will also review participant's medical records, if available, for care relating to sexual health such as HIV/STI testing and results, PrEP use, and risk factors starting from study enrollment onward.

C.10. Design Considerations.
The study team thought carefully about our design considerations regarding the collection of biological specimens for either HIV or other related STIs, but decided against it for two reasons: (1) from our experience in the field, we believe the incidence and prevalence rates of both HIV and STIs will be relatively low in this young age group, making testing not particularly helpful for either main or secondary outcomes, and (2) in certain geographic regions, such as the South, that adding a specimen collection might make it more difficult to obtain a waiver of parental consent for participation in this research, which we believe is of paramount importance. In addition, given that HIV/STI testing will be addressed in MyPEEPS Mobile intervention, and is an outcome of interest, we will not provide HIV/STI testing for study participants. Instead all study participants will be provided access to HIV/STI testing resources at the study endpoint. Importantly, participants are expected to continue normal healthcare activities during the course of the study and will be offered HIV/STI testing referral information.

In addition to HIV/STI testing referral information, if during the course of participation, study staff identifies any young person who screens for moderate/high levels of substance use or mental health problems, they will be referred to local providers of care as well as to the TREVOR hotline if appropriate. Study staff will use screening instruments that are used commonly in adolescent primary care. If a young person reports abuse either sexual or otherwise, we will screen for imminent risk and make a report of the abuse according to state-specific rules. We will do a "well-being" de-briefing with all participants at the end of each visit. If they report being upset, we will screen for imminent risk of suicidality. We will not ask specifically about criminal behavior, e.g., stealing, vandalism, etc.

Table 3. Outcome Measures

	Pilot Study		Multi-Site RCT – Timepoints (months)				
	Pre-Test	Post-Test	Baseline	3	6	9	12
**Demographics**							
Sociodemographic: (e.g., age, race/ethnicity level of education, housing status)	▪		▪				
Technology Assessment	▪		▪				
Functional Health Literacy[88]	▪		▪				
Information Privacy Concerns[89]	▪		▪				
**Primary Outcome Measures**							
Sexual risk behaviors (adapted from AIDS-Risk Behavior Assessment)[90-92]	▪	▪	▪	▪	▪	▪	▪
nPEP and PrEP Use (if guidelines change for under 18 years)	▪	▪	▪	▪	▪	▪	▪
HIV and STI Testing	▪	▪	▪	▪	▪	▪	▪
**Intermediate Outcome Measures**							
Self-efficacy for safer sex and situational temptation for unsafe sex[93]	▪	▪	▪	▪	▪	▪	▪
Condom Errors[94]	▪	▪	▪	▪	▪	▪	▪
**Secondary Outcomes – Behavioral Intention**							
Intent to have anal sex	▪	▪	▪	▪	▪	▪	▪
Intent to use condoms during anal sex	▪	▪	▪	▪	▪	▪	▪
Intent to uptake PrEP/nPEP (if guidelines change for	▪	▪	▪	▪	▪	▪	▪

under 18 years)							
**Program Evaluation (* = intervention; ✓ = delayed intervention)**							
Knowledge of and beliefs about the content of the MyPEEPS Mobile		▪		*			✓
MyPEEPS Intervention Acceptability and Tolerability[95]		▪		*			✓
Information System Success[96]		▪		*			✓

C.11. Study Outcomes.

Our outcome measures have been used in our MyPEEPS pilot study with YMSM (R34MH079707; PI: Garofalo). **We have 4 levels of outcomes:** primary, secondary, intermediate and program evaluation. A detailed list of outcome measures within each category and the time points when they will be collected is listed in Table 3. We also detail the primary and intermediate outcome measures below.

C.11.a. Primary Outcome Measures.

Male-male sexual risk in the prior 3-month period will be evaluated at all-time points using the AIDS-Risk Behavior Assessment (ARBA) adapted for YMSM.[90-92] Variables of interest include: Our primary outcomes will be: total number of sex partners and condomless anal sex partners, frequencies for condomless anal sex acts, anal sex with and without condoms under the influence of alcohol/drugs, nPEP and PrEP (if FDA-approved for this age group) use and self-reported HIV/STI testing.

C.11.b. Secondary Outcomes.

Measures are related to behavioral intention. In the case of our study, the secondary outcome measures will focus on intent to reduce the male-male sexual risk behaviors outlined above.

C.11.c. Intermediate Outcome Measures.

Behavior change is moderated and mediated by intermediate personal and social factors including knowledge (e.g., how to use a condom), self-efficacy, and inter-personal communication skills.[97] Internalized homophobia has also been associated with sexual risk behavior in YMSM.[98] This study is not powered for tests of moderation and mediation, nonetheless the purpose of the inclusion of these factors is to measure the impact of the intervention on them as intermediate targets given their potential role in behavior change.

Self-efficacy for safer sex and situational temptation for unsafe sex. The 10-item scale[93] (α= 0.72) was used to assess self-efficacy in practicing condom use and safer sex communication with a partner. A sample item is "*If I didn't want to have sex with my partner, I would be able to say 'no.'*"

Condom errors. The 12-item Questionnaire[94] was adapted to reflect a 6-week recall period (α= .60). A sample question is "*When you used condoms during the last 6 weeks, how often was the condom put on the wrong side up so that it had to be flipped over?*"

Demographic characteristics. Sociodemographic data of study participants will be collected at baseline, including age, race/ethnicity, sexual orientation, highest level of education, and housing status. We will also collect dating behavior, health literacy, depression, substance use, HIV knowledge, internalized homonegativity, and technology use data.[98-101]

We have selected our outcome measures based on our past work and specifically our successful MyPEEPS pilot trial. If NIMHD seeks to harmonize the outcomes across all of the U01 projects then we will work with the agency to include other measures or adapt our existing measures.

C.12. COVID-19 Measures.

The investigative team decided to temporarily attach a COVID-19 related block of questions to the MyPEEPS RCT 3-, 6-, 9-, and 12-month follow-up surveys to assess the impacts of the outbreak. The question block will be temporarily attached at the end of all the follow up surveys for the next 3 months, i.e, through the end of August. That should result in getting everyone who

hasn't completed the study at their next visit. If COVID impacts continue through fall of 2020, the investigative team will reassess the need to continue the deployment of this survey block beyond the initial timeframe (end of August). The purpose of this assessment is to better understand the impact of COVID-19 on the current RCT study outcomes specifically as it relates to sexual risk behaviors, PrEP use and HIV/STI testing. The block will include 27 questions concerning: MROS3; Shelter in Place/Stay at Home Orders and Voluntary Self-Quarantine; HIV/Testing During COVID-19 Outbreak; PrEP Use and Refill Access During COVID-19 Outbreak; Risk Behaviors During COVID-19 Outbreak.

C.13. Sound Retention Efforts.

Participants will be asked at the end of the screening what would be the best way for us to remind them of the appointment (voice phone, text, e-mail). We will explain to participants that in all cases a clear boundary exists between the research project and agency staff that prevents the sharing of information we learn about them during the research study with anyone at the agency. Participant retention during the intervention will be enhanced in several ways. First, participants will be compensated $25 for their time to complete the baseline assessment, up to $100 for completing all of the MyPEEPS modules, and additional compensation for completing each of the follow-up assessments. Participants will be compensated $30 for completing the 3 month survey, $35 for completing the 6 month participants will be compensated $45 for completion of the 12 month survey. In addition, at each followup visit (3, 6, 9 and 12 months), participants will be asked to update their locator information.

Contact (phone calls, text, email or note as preferred by the participant) will be made by the site study staff prior to the follow-up visit to confirm or reschedule an appointment. The discrete contact will thank the participants for being in the program, remind them of the date and time of their next follow-up appointment and note the telephone number that can be called if rescheduling is necessary. Participants who do not respond and cannot be located during the acceptable window period are coded as a missed assessment and contact will continue for the next assessment. We will contact participant until they expresses the desire to be dropped from the study. If a participant moves away from the original project conducted or arrangements will be made to transfer their contact information to a different site. These extensive procedures will be used to promote participant attendance at the follow-up visits and has been shown to be successful in previous intervention trials.[102] Using the following sound retention efforts as well as reimbursement for completion of surveys, we are confident that we will retain at least 80% of our study sample at a single follow-up assessment for each study arm.[86] We have methods in place to maximize intervention completion including: 1) gaming components of our intervention, 2) monetary compensation for completion of intervention modules, and 3) automated electronic (e-mail/ text) reminders.

C.14. Statistical Procedures:

All multivariate analyses will be preceded by standard descriptive bivariate analyses to describe key variables and relationships among key variables. These analyses will include means, frequency tables, histograms, and examination of distributions. All statistical tests will be two-sided tests with the level of significance at 0.05 (PRS Efficacy Criteria for Best Evidence Risk Reduction Individual Level –Tier 1).[86] Outcomes: This study will examine whether the intervention will (1) reduce the number of sex partners, number of anal sex partner with whom a condom was not used, etc.; and (2) increase the proportion of anal sex with condom use, etc. We will also examine whether the intervention will (3) be still effective (i.e., the outcomes are not getting worse) during 3 and 6 months follow up period. Primary Outcomes: There are two types of primary outcome variables in this study: (1) counts, (i.e., number of sex partners, number of anal sex partners with whom a condom was not used, etc.) and (2) binary/binomial - percentages, (i.e. percentage of anal sex acts) (Table 3 – outcome variables). We assume that all count outcome variables follow Poisson or negative binomial (NB) distribution, $y \sim Poi(\lambda)$ or $y \sim NB(\lambda, \kappa)$, and all percentage outcomes follow binomial distribution, $y \sim bin(n, p)$. Let the expected value of outcome, $E(y) = \varphi$, so $\varphi = \lambda$ for count outcomes and $\varphi = p$

for percentage outcomes. We propose the following individual growth model, a special case of generalized linear mixed model (GLMM), to analyze both count and percentage outcomes. The level of analysis will be assessed at each data point. For person i and data assess wave W:

$$h(\varphi_{i,w}) = \beta_{oi} + \beta_{1i}Arm + \beta_{2i}W + \beta_{3i}I + \beta_{4i}FU + \beta_{5i}X,$$
$$B_{ij} = \eta_{i0} + \eta_{i1}COV + \mu_{ij}, \qquad j = 0,1,2,3,4, and\ 5$$

Where $h(.)$ is the link function for the GLMM. We will use log-links (Poisson or Negative Binomial regression) for count outcomes and logit-links (logistic regression) for percentage outcomes. $\mu_{ji} iid{\sim}N(0, \sigma_j^2)$ are personal level random effects. Variables Arm, W, I, and FU are design variables. The codes for the design variables are presented in Table 4. Variable Arm is the indicator for intervention arms (0=intervention arm and 1=delayed intervention arm); variable W is data assess wave indicator (0=baseline, 1=3 months, 2=6 months, etc.); variable I is the intervention indicator (0=pre- intervention, and 1=post-intervention); variable FU is the indicator for follow up time period (1=3 month follow up, 2=6 month follow up, and 0=otherwise). Variable COV is a vector of personal level covariates at the baseline: it includes study site (Birmingham, Chicago, New York, Seattle), age, recruitment method (in-person vs online), primary race/ethnicity (Blacks/African Americans, Hispanics/Latinos, American Indians/Alaska Natives, Asian Americans, Native Hawaiians and Other Pacific Islanders), self-identified as gay/homosexual (bisexual/other), self-reported baseline HIV serostatus, etc. Variable $\boldsymbol{XX}$ is a vector of time- dependent covariates assessed at each wave of data for each subject, such as STD and HIV tests, knowledge of prevention messages, etc. This model will include both baseline personal level covariates as well as personal-wave level covariates in order to control for different types of potential confounders.

Table 4. Codes for design variables

Months	Intervention Arm				Delayed Intervention Arm			
	Arm	W	I	FU	Arm	W	I	FU
Baseline	0	0	0	0	1	0	0	0
3 mo	0	1	1	0	1	1	0	0
6 mo	0	2	1	1	1	2	0	0
9 mo	0	3	1	2	1	3	0	0
12 mo	-	-	-	-	1	4	1	0

Regression parameter β_3 is the mean value change for the outcome variables between pre and post intervention, is the main outcome measurement, and measures the impact of the intervention on each outcome is the mean value change for the outcome variables between pre and post intervention, is the main outcome measurement, and measures the impact of the intervention on each outcome variable. For the Poisson or Negative Binomial models, $\exp(\beta_3)$ is the ratio of mean counts (i.e., risk ratio) between post and pre intervention. For the logistic models, $\exp(\beta_3)$ is the odds ratio between post and pre intervention. Regression parameter β_4 is the rate of mean value change for the outcome values during the follow up period. Therefore, we will use superiority tests instead of traditional comparative tests.

We propose a GLMM to analyze data. One of the main advantages of GLMM is to provide unbiased estimates when there are missing outcomes during the follow up period. For the missing values at the baseline or partial baseline collected data, we will use a multiple imputation approach.[131] Models will also be run on the raw, non-imputed data. Inferences for the trial arm, wave, and interaction between trial arm and wave did not differ between the analyses of the raw and multiply imputed data. Rates of reduction will be calculated from population-averaged rates, which control for all other covariates in the multivariable model. Models will be calculated using R, and model fit will be evaluated by diagnostic statistics and residual plots. Secondary Outcomes: We will use the proposed GLMM to analyze secondary outcomes with appropriate choice of link functions according to the outcomes. All analyses will be done on the final data set (baseline n=900).

C.14.a. Missing data.

Missing data may occur in the proposed study in several ways. First, missing data may occur due to item non-response. When missing data is limited to only a few items on a measure, we will prorate

total scores for a measure by taking an average score on the measure and multiplying it by the total number of items in the scale. Missing data can also occur from attrition due to missed assessments or dropout from the study. Prior to performing any outcome analyses, we will evaluate the amount, reasons, and patterns of missing data. If the reason for missing data is not related to the outcome of interest, then the missing data are considered to be missing completely at random (MCAR) and complete case analysis will still generate unbiased estimates.[103] We will conduct sensitivity analyses to compare estimates of treatment effects with and without multiple imputation to assess the effect of missing data on statistical inference.

C.15. MyPEEPS Mobile RCT In-depth Interviews

Selected participants will be interviewed about the MyPEEPS Mobile RCT to better understand their overall experience using the MyPEEPS application and the study recruitment process.

Recruitment: For the in-depth interviews, we will recruit up to 40 participants across the four study sites (New York, Chicago, Birmingham, and Seattle), 10 participants from each study site. We anticipate that a total of 40 participants will be an adequate number of participants to reach saturation based on our earlier studies focusing on similar topics.[32] Through a purposive sampling we will draw from a group of participants who agreed to be contacted for future studies and completed the MyPEEPS mobile RCT. Participants will be given information about the in-depth interview, the time involved (approximate length of time: 1-1.5 hour), and compensation ($30). The interview compensation is consistent with payments made in prior studies.

Procedures: All in-depth interviews will be conducted with participants individually and recorded using an online platform (e.g. Zoom). Before the start of the interview, the study staff will first explain the purpose of the project and participants will review and sign a written informed assent/consent in electrotonic form. The informed assent/consent form provides details of the study procedures, risks, benefits, site contact information, and the nature of confidentiality and voluntary participation. The consent process also covers information on the timeline, task involved, and compensation for time. To ensure understandability, the study staff will read the form to the participant and ask them to summarize their understanding of the study procedures. Before a participant signs the informed assent/consent form, staff will answer any questions. Participants will be given a copy of the informed assent/consent form for their records.

Following the completion of the electronic informed assent/consent process, participants will begin the indepth interview. We will ask structured questions pertaining to the participants' overall experience with the MyPEEPS Mobile RCT and the recruitment process, and the relevance or impact of the MyPEEPS application to their lives. During the interview, we will also provide participants with a handout listing the MyPEEPS activities to help with recall. The individual in-depth guide will include the following questions

1) Please describe your experience navigating, or moving through, the MyPEEPS app activities and any technical issues.
2) What are some changes you think should be made to the MyPEEPS mobile app to make it easier to use? What worked/ did not work? What app activities or topics would you add or change? What would you keep?
3) How long did it take you to complete each app activity? How did this fit into your lifestyle and schedule?
4) At the completion of this study, would you want to keep using the MyPEEPS app? Why or why not?
5) Thinking back on the information you learned from the MyPEEPS app, how would you apply this information/lesson/activity in your own life? Have your health behaviors changed because of using the MyPEEPS app? If so, how/ what has changed in your health behaviors?

6) How do the MyPEEPS activities reflect your cultural beliefs, norms, and values and how were the MyPEEPS activities relevant or irrelevant to your sexual health or health overall?
7) How would you modify the outreach and enrollment to improve the process or make it easier?
8) How comfortable were you with the online recruitment and enrollment process?
9) Would you prefer the recruitment and enrollment process to be online or in-person? Please explain why.
10) Please describe what kind of access to HIV prevention information or care you have in your community or school? Do you feel like obtaining HIV prevention information is difficult for you? If so, please explain?

We will also ask probing questions to stimulate discussion. Furthermore, the guide will be informed by the feedback and commentary from members of our investigative team who have extensive clinical and research experience working with our study population. During the interview, the interviewer will also take notes.

Transcription: All individual interview audio recording will be transcribed verbatim through a transcription company. Any identifiers will be deleted from the audio and removed from the transcript. The team will adhere to qualitative processes to ensure the credibility, conformability, and dependability, and transferability of the qualitative data from theses analyses. To support the credibility use “member checks,” i.e., sharing of initial data interpretations with participants, to ensure accurate interpretations. Triangulation of findings, along with reflexivity, will enhance the confirmability of the interpretations. The investigators will carefully record an audit trail and keep extensive field notes to facilitate transferability of study findings into other contexts (Guba, 1981). Transcripts will be analyzed independently for content by research team members in these methods.

Data Analysis: The research team will adhere to qualitative research processes to ensure the credibility, confirmability, dependability and transferability of the qualitative data from these analyses. The study team will meet and review transcripts and notes. Drs. Schnall and Hidalgo[17,21,62-64] who both have experience in qualitative analysis will work with two research team members to code the transcripts. Field notes and transcripts will be analyzed by the researchers using NVivo™ (QSR International, Victoria, Australia) software. Participants’ statements will be captured using memoing and then sorted into the categories of interest or descriptive thematic categories. Open coding will be used to develop initial data categories. Some codes will be derived from the questions included in the interview and other codes will emerge from themes and patterns identified in the narratives. An initial set of codes will be independently generated by two coders. Codes will then be compared and synthesized to result in shared coding categories and subcategories, all with definitions, inclusion and exclusion criteria, and examples. Coders will discuss discrepancies until they reach consensus. The research team will meet and review transcripts and notes. The goal is to collect feedback about the participants’ overall experience with the MyPEEPS mobile RCT, technological difficulties, recruitment, and to identify subject matter that should be included to enhance the MyPEEPS mobile and recruitment process for future studies.

D. Assess the feasibility of using In-home HIV tests for assessing HIV status in a sample of very YMSM by conducting follow-up testing with MyPEEPS RCT participants.

Goals: Assess the feasibility of using the HIV home test for assessing HIV status in a sample of very YMSM.

Design Overview**.** In-home HIV testing will be conducted with MyPEEPS RCT participants 3 months following their completion of the trial. Participants will complete an in-home HIV test and an online survey that includes questions about demographic characteristics, health literacy, mental health and substance use, sexual risk behaviors, HIV testing history, and opinions regarding HIV testing.

Recruitment. We will recruit participants 3 months after they complete the MyPEEPS RCT. Participants will be contacted by study staff using their preferred method of communication to determine interest in the study. All participants within the recruitment timeframe (3 months post-RCT) will be approached except those withdrawn from the trial. Participants who voluntarily disclose to study staff while recruiting that they tested positive for HIV (seroconverted) when they were in the trial or since their participation ended will not be recruited in the current study but will be offered resources to be linked to care, if not already linked.

Sample. Our sample will include participants enrolled into the trial (inclusive of YMSM, Nonbinary individuals, and YTGW [who transitioned during or after the study], aged 13-21).

Eligibility criteria. In addition to being a MyPEEPS RCT participant, we will also include the following eligibility criteria: 1) understand the limitations of the OraQuick test (e.g., confirmatory test is needed); 2) not having tested positive for HIV since enrollment in the trial.

Study Enrollment. Study participants will conduct an enrollment visit remotely to provide informed consent (+17) or assent (13-16), enroll and complete provide an ‘Address Survey’ to provide a mailing address so study staff can send study materials needed for the baseline study visit. Remote informed consent will be conducted via video conferencing such as Skype, Zoom, FaceTime using REDCap to collect e-consent or e-assent and answer participant questions for the Address Survey. Through the REDCap platform, participants will be able to securely download a copy of the e-consent or e-assent for their records. As part of the REDCap e-consent/e-assent platform, the electronic forms will be automatically archived and securely stored in REDCap. Prior to the enrollment visit, those providing informed assent will be asked to have a parent or guardian provide e-consent to participate in the research. Parents/guardians will be able to provide e-consent through the REDCap platform and securely download a copy of the e-consent for their records. Those who secure parental/guardian consent will be asked in the Address Survey to confirm their parent/guardian’s submitted consent and that they are aware of the materials that will be mailed to their home. All participants will complete an HIV Home Testing Understanding Assessment with study staff to ensure participants fully comprehend how use the OraQuick rapid test prior to receiving it. Following enrollment, participants will be sent a study package with the following materials: an OraQuick in-home rapid HIV test kit, condoms, and study information listing the study team’s contact information. OraQuick tests will be sent via tracked shipping through FedEx. During the remote baseline visit, participants will join via video conferencing to complete an in- home HIV test and an online survey that includes questions about demographic characteristics, health literacy, mental health and substance use, sexual risk behaviors including number of individuals they engaged in anal or oral sex with, condomless intercourse, as well as their HIV testing history, and opinions regarding HIV testing. The baseline survey will also include questions on PEP/PrEP use and adherence, drug and alcohol use, and the HIV Risk Index. The survey will also ask questions to assess the impacts of the COVID-19 outbreak. Remote participants who test negative (non-reactive) will receive the HIV prevention, PrEP assessment and referral information via a secure e-email reviewed during the study visit video call. Remote participants who receive a preliminary positive result will be linked to care. Remote in-home HIV testing at baseline will use the OraQuick in-home tests. Participants will be shown how to conduct the test via video conference call. If a participant is found to be preliminary positive (reactive) during the baseline testing, they will be linked to care at a health clinic or hospital closest to them or a healthcare provider of their choice. If they are non-reactive, the study staff will wrap up the study visit. Study staff will record participant’s HIV test results in their own individual REDCap record.

REFERENCES

1. Purcell DW, Johnson CH, Lansky A, et al. Estimating the population size of men who have sex with men in the United States to obtain HIV and syphilis rates. *Open AIDS J.* 2012;6:98-107.
2. Center for Disease and Prevention. HIV Among Gay and Bisexual Men. 2014, 2014.
3. Center for Disease and Prevention. Diagnoses of HIV infection and AIDS among adolescents and young adults in the United States and 5 U.S. dependent areas, 2006-2009. 2012.
4. Center for Disease and Prevention. New HIV infections in the United States, 2007-2010. 2010, 2015.
5. Hidalgo MA, Kuhns LM, Hotton AL, Johnson AK, Mustanski B, Garofalo R. The MyPEEPS randomized controlled trial: a pilot of preliminary efficacy, feasibility, and acceptability of a group-level, HIV risk reduction intervention for young men who have sex with men. *Arch Sex Behav.* 2015;44(2):475-485.
6. Lenhart A. *Teens, Social Media & Technology Overview 2015.* Pew Research Center: Internet, Science & Tech;2015.
7. Chiasson MA, Parsons JT, Tesoriero JM, Carballo-Dieguez A, Hirshfield S, Remien RH. HIV behavioral research online. *J Urban Health.* 2006;83(1):73-85.
8. Pequegnat W, Rosser BR, Bowen AM, et al. Conducting Internet-based HIV/STD prevention survey research: considerations in design and evaluation. *AIDS Behav.* 2007;11(4):505-521.
9. Stall R, van Griensven F. New directions in research regarding prevention for positive individuals: questions raised by the Seropositive Urban Men's Intervention Trial. *AIDS.* 2005;19 Suppl 1:S123-127.
10. Rosser BR, Oakes JM, Horvath KJ, Konstan JA, Danilenko GP, Peterson JL. HIV sexual risk behavior by men who use the Internet to seek sex with men: results of the Men's INTernet Sex Study-II (MINTS-II). *AIDS Behav.* 2009;13(3):488-498.
11. Wolitski RJ, Gómez CA, Parsons JT. Effects of a peer-led behavioral intervention to reduce HIV transmission and promote serostatus disclosure among HIV-seropositive gay and bisexual men. *AIDS.* 2005;19 Suppl 1:S99-109.
12. Stockwell MS, Westhoff C, Kharbanda EO, et al. Influenza vaccine text message reminders for urban, low-income pregnant women: a randomized controlled trial. *Am J Public Health.* 2014;104 Suppl 1:e7-12.
13. Stockwell MS, Kharbanda EO, Martinez RA, Vargas CY, Vawdrey DK, Camargo S. Effect of a text messaging intervention on influenza vaccination in an urban, low-income pediatric and adolescent population: a randomized controlled trial. *JAMA.* 2012;307(16):1702-1708.
14. Stockwell MS, Hofstetter AM, DuRivage N, et al. Text message reminders for second dose of influenza vaccine: a randomized controlled trial. *Pediatrics.* 2015;135(1):e83-91.
15. Schnall R, Wantland D, Velez O, Cato K, Jia H. Feasibility testing of a web-based symptom self-management system for persons living with HIV. *J Assoc Nurses AIDS Care.* 2014;25(4):364-371.
16. Schnall R, Rojas M, Travers J, Brown W, Bakken S. Use of Design Science for Informing the Development of a Mobile App for Persons Living with HIV. *AMIA Annu Symp Proc.* 2014;2014:1037-1045.
17. Schnall R, Bakken S, Rojas M, Travers J, Carballo-Dieguez A. mHealth Technology as a Persuasive Tool for Treatment, Care and Management of Persons Living with HIV. *AIDS Behav.* 2015;19 Suppl 2:81-89.
18. Aliabadi N, Rojas M, Carballo- Diéguez A. Informing the Development of a Mobile App for HIV Prevention in High-Risk, Urban Men who Have Sex with Men (MSM). American Public Health Association, 2014; 2014; New Orleans, LA.
19. Aliabadi N, Carballo-Dieguez A, Bakken S, et al. Using the Information-Motivation-Behavioral Skills Model to Guide the Development of an HIV Prevention Smartphone Application for High-Risk MSM. *AIDS Educ Prev.* 2015;27(6):522-537.
20. Schnall R, Sperling JD, Liu N, Green RA, Clark S, Vawdrey DK. The effect of an electronic "hard-stop" alert on HIV testing rates in the emergency department. *Stud Health Technol Inform.* 2013;192:432-436.
21. Schnall R, Smith AB, Sikka M, et al. Employing the FITT framework to explore HIV case managers' perceptions of two electronic clinical data (ECD) summary systems. *Int J Med Inform.* 2012;81(10):e56-62.
22. Ventuneac A, Carballo-Diéguez A, Leu CS, et al. Use of a rapid HIV home test to screen sexual partners: an evaluation of its possible use and relative risk. *AIDS Behav.* 2009;13(4):731-737.
23. Schnall R, Carballo-Diéguez A, Larson E. Can the HIV home test promote access to care? Lessons learned from the in-home pregnancy test. *AIDS Behav.* 2014;18(12):2496-2498.
24. Nodin N, Carballo-Diéguez A, Ventuneac AM, Balan IC, Remien R. Knowledge and acceptability of alternative HIV prevention bio-medical products among MSM who bareback. *AIDS Care.* 2008;20(1):106-115.
25. Martinez O, Wu E, Shultz AZ, et al. Still a hard-to-reach population? Using social media to recruit Latino gay couples for an HIV intervention adaptation study. *J Med Internet Res.* 2014;16(4):e113.
26. Carballo-Dieguez A, Dolezal C, Nieves-Rosa L, Diaz F. Similarities in the Sexual Behavior and HIV Risk Factors of Colombian, Dominican, Mexican, and Puerto Rican MSM Residing in New York City. *null.* 2001;12(4):49-67.
27. Carballo-Diéguez A, Dolezal C, Leu CS, et al. A randomized controlled trial to test an HIV-prevention intervention for Latino gay and bisexual men: lessons learned. *AIDS Care.* 2005;17(3):314-328.
28. Carballo-Diéguez A, Bauermeister JA, Ventuneac A, Dolezal C, Balan I, Remien RH. The use of rectal douches among HIV-uninfected and infected men who have unprotected receptive anal intercourse: implications for rectal

microbicides. *AIDS Behav.* 2008;12(6):860-866.
29. Carballo-Diéguez A, Ventuneac A. Prioritization of HIV Prevention over Other Pressing Concerns. In:2007.
30. Schnall R, Odlum M, Gordon P, Bakken S. Barriers to implementation of a Continuity of Care Record (CCR) in HIV/AIDS care. *Stud Health Technol Inform.* 2009;146:248-252.
31. Schnall R, Kaufman D, Hyun S, Bakken S. Cognitive Evaluation of Case Managers' Use of a Continuity of Care Document for Persons Living with HIV. AMIA Annual Symposium, 2009; 2009; San Francisco, CA.
32. Schnall R, Gordon P, Camhi E, Bakken S. Perceptions of factors influencing use of an electronic record for case management of persons living with HIV. *AIDS Care.* 2011;23(3):357-365.
33. Schnall R, Cimino JJ, Currie LM, Bakken S. Information needs of case managers caring for persons living with HIV. *J Am Med Inform Assoc.* 2011;18(3):305-308.
34. Schnall R, Cimino JJ, Bakken S. Development of a prototype continuity of care record with context-specific links to meet the information needs of case managers for persons living with HIV. *Int J Med Inform.* 2012;81(8):549-555.
35. Schnall R, Bakken S. Testing the Technology Acceptance Model: HIV case managers' intention to use a continuity of care record with context-specific links. *Inform Health Soc Care.* 2011;36(3):161-172.
36. Kukafka R, Millery M, Chan C, LaRock W, Bakken S. Assessing the need for an online decision-support tool to promote evidence-based practices of psychosocial counseling in HIV care. *AIDS Care.* 2009;21(1):103-108.
37. Gordon P, Camhi E, Hesse R, et al. Processes and outcomes of developing a continuity of care document for use as a personal health record by people living with HIV/AIDS in New York City. *Int J Med Inform.* 2012;81(10):e63-73.
38. Bakken S, Holzemer WL, Portillo CJ, Grimes R, Welch J, Wantland D. Utility of a standardized nursing terminology to evaluate dosage and tailoring of an HIV/AIDS adherence intervention. *J Nurs Scholarsh.* 2005;37(3):251-257.
39. Sheehan B, Lee Y, Rodriguez M, Tiase V, Schnall R. A comparison of usability factors of four mobile devices for accessing healthcare information by adolescents. *Appl Clin Inform.* 2012;3(4):356-366.
40. Schnall R, Rojas M, Bakken S, et al. A user-centered model for designing consumer mobile health (mHealth) applications (apps). *J Biomed Inform.* 2016;60:243-251.
41. Brown W, Yen PY, Rojas M, Schnall R. Assessment of the Health IT Usability Evaluation Model (Health-ITUEM) for evaluating mobile health (mHealth) technology. *J Biomed Inform.* 2013;46(6):1080-1087.
42. Arya M, Kumar D, Patel S, Street RL, Giordano TP, Viswanath K. Mitigating HIV health disparities: the promise of mobile health for a patient-initiated solution. *Am J Public Health.* 2014;104(12):2251-2255.
43. Center for Disease and Prevention. DHAP HIV Funding Awards by State and Dependent Area (Fiscal Year 2012). 2012, 2015.
44. Simoni JM, Huh D, Frick PA, et al. Peer support and pager messaging to promote antiretroviral modifying therapy in Seattle: a randomized controlled trial. *J Acquir Immune Defic Syndr.* 2009;52(4):465-473.
45. Nelson KM, Simoni JM, Pearson CR, Walters KL. 'I've had unsafe sex so many times why bother being safe now?': the role of cognitions in sexual risk among American Indian/Alaska Native men who have sex with men. *Ann Behav Med.* 2011;42(3):370-380.
46. New York City Department of Health & Mental Hygiene. *New York City HIV/AIDS Annual Surveillance Statistics.* 2014.
47. Sisk R. South Bronx is poorest district in nation, U.S. Census Bureau finds: 38% live below poverty line. *NY Daily News*2010.
48. New York City Department of Health & Mental Hygiene. *The Bronx Knows HIV Testing Initiative Final Report.* 2011.
49. US Census Bureau. State & County QuickFacts. 2015; http://quickfacts.census.gov/qfd/states/17/1714000.html, 2015.
50. Chicago Department of Health. *HIV/STI Surveillance Report, Chicago.* 2014.
51. Alabama Department of Health. *HIV Surveillance Annual Report, Alabama.* 2013.
52. US Census Bureau. Quick Facts: Seattle (city). 2015; http://quickfacts.census.gov/qfd/states/53/5363000.html, 2015.
53. King County Department of Health. *HIV/AIDS Surveillance Report for Seattle and King County.* 2015.
54. Raghavendran B. High rate of homelessness among King County's LGBTQ youth. *Seattle Times*2015.
55. Schueller SM, Begale M, Penedo FJ, Mohr DC. Purple: a modular system for developing and deploying behavioral intervention technologies. *J Med Internet Res.* 2014;16(7):e181.
56. Brennan J, Kuhns LM, Johnson AK, et al. Syndemic theory and HIV-related risk among young transgender women: the role of multiple, co-occurring health problems and social marginalization. *Am J Public Health.* 2012;102(9):1751-1757.
57. Garofalo R, Deleon J, Osmer E, Doll M, Harper GW. Overlooked, misunderstood and at-risk: exploring the lives and HIV risk of ethnic minority male-to-female transgender youth. *J Adolesc Health.* 2006;38(3):230-236.
58. Bouris A, Voisin D, Pilloton M, et al. Project nGage: Network Supported HIV Care Engagement for Younger Black Men Who Have Sex with Men and Transgender Persons. *J AIDS Clin Res.* 2013;4.

59. Hotton AL, Garofalo R, Kuhns LM, Johnson AK. Substance use as a mediator of the relationship between life stress and sexual risk among young transgender women. *AIDS Educ Prev.* 2013;25(1):62-71.
60. Kuhns LM, Reisner SL, Mimiaga MJ, Gayles T, Shelendich M, Garofalo R. Correlates of PrEP Indication in a Multi-Site Cohort of Young HIV-Uninfected Transgender Women. *AIDS Behav.* 2016;20(7):1470-1477.
61. Garofalo R, Johnson AK, Kuhns LM, Cotten C, Joseph H, Margolis A. Life skills: evaluation of a theory-driven behavioral HIV prevention intervention for young transgender women. *J Urban Health.* 2012;89(3):419-431.
62. Hidalgo MA, Cotten C, Johnson AK, Kuhns LM, Garofalo R. 'YES, I AM MORE THAN JUST THAT': GAY/BISEXUAL YOUNG MEN RESIDING IN THE UNITED STATES DISCUSS THE INFLUENCE OF MINORITY STRESS ON THEIR SEXUAL RISK BEHAVIOR PRIOR TO HIV INFECTION. *Int J Sex Health.* 2013;25(4):291-304.
63. Schnall R, Clark S, Olender S, Sperling JD. Providers' perceptions of the factors influencing the implementation of the New York State mandatory HIV testing law in two Urban academic emergency departments. *Acad Emerg Med.* 2013;20(3):279-286.
64. Schnall R, Rojas M, Travers J. Understanding HIV testing behaviors of minority adolescents: a health behavior model analysis. *J Assoc Nurses AIDS Care.* 2015;26(3):246-258.
65. US Department of Health & Human Services. Usability.gov. 2015, 2015.
66. Nielsen J, Molich R. Heuristic evaluation of user interfaces. Proceedings of the SIGCHI Conference on Human Factors in Computing Systems; 1990; Seattle, Washington, USA.
67. Lee Y, Johnson S, Trembowelski S, Schnall R, Sheehan R, Bakken S. Cognitive walkthrough evaluation of an investigator profiles directory. Proc AMIA Annu Fall Symp 2011; 2011.
68. Schnall R, Wantland D, Velez O, Yen P, Cato K, Bakken S. Development and Testing of a web-based symptom self-management Tool for PLWH. International Congress on Nursing Informatics; 2012; Montreal, Canada.
69. Bertini E, Gabrielli S, Kimani S. *Appropriating and assessing heuristics for mobile computing.* 2006.
70. TechSmith. Morae Usability and Web Site Testing. 1995; http://www.techsmith.com/products/morae/default.asp, 2005.
71. Gibson L, Gaffey E, Spendlove J. The usability factor: more than just a pretty interface. 8th International Congress in Nursing Informatics; 2003; Rio de Janeiro, Brazil.
72. Nielson J. Ten Usability Heuristics. In:2005.
73. Bright TJ, Bakken S, Johnson SB. Heuristic evaluation of eNote: an electronic notes system. *AMIA Annu Symp Proc.* 2006:864.
74. Nielson J. Heuristic Evaluation. In: Nielson J, ed. *Usability Inspection Methods.* John Wiley & Sons; 1994.
75. Faulkner L. Beyond the five-user assumption: benefits of increased sample sizes in usability testing. *Behav Res Methods Instrum Comput.* 2003;35(3):379-383.
76. Yen PY, Wantland D, Bakken S. Development of a Customizable Health IT Usability Evaluation Scale. *AMIA Annu Symp Proc.* 2010;2010:917-921.
77. Davis FD. Perceived Usefulness, Perceived Ease of Use, and User Acceptance of Information Technology. *MIS Quarterly.* 1989;13(3):319-340.
78. Wyatt JC. When to use web-based surveys. *J Am Med Inform Assoc.* 2000;7(4):426-429.
79. Grella CE, Scott CK, Foss MA, Dennis ML. Gender similarities and differences in the treatment, relapse, and recovery cycle. *Eval Rev.* 2008;32(1):113-137.
80. Scott CK, Sonis J, Creamer M, Dennis ML. Maximizing follow-up in longitudinal studies of traumatized populations. *J Trauma Stress.* 2006;19(6):757-769.
81. Lloyd CE, Johnson MR, Mughal S, et al. Securing recruitment and obtaining informed consent in minority ethnic groups in the UK. *BMC Health Serv Res.* 2008;8:68.
82. Altman DG, Bland JM. Statistics notes. Treatment allocation in controlled trials: why randomise? *BMJ (Clinical research ed).* 1999;318(7192):1209.
83. Hedden SL, Woolson RF, Malcolm RJ. Randomization in substance abuse clinical trials. *Subst Abuse Treat Prev Policy.* 2006;1:6.
84. Schulz KF, Altman DG, Moher D, Group C. CONSORT 2010 Statement: Updated guidelines for reporting parallel group randomised trials. *J Clin Epidemiol.* 2010;63(8):834-840.
85. Vickers AJ. How to randomize. *J Soc Integr Oncol.* 2006;4(4):194-198.
86. Lyles CM, Crepaz N, Herbst JH, Kay LS, Team HAPRS. Evidence-based HIV behavioral prevention from the perspective of the CDC's HIV/AIDS Prevention Research Synthesis Team. *AIDS Educ Prev.* 2006;18(4 Suppl A):21-31.
87. Suresh K. An overview of randomization techniques: An unbiased assessment of outcome in clinical research. *J Hum Reprod Sci.* 2011;4(1):8-11.
88. Baker DW, Williams MV, Parker RM, Gazmararian JA, Nurss J. Development of a brief test to measure functional health literacy. *Patient Educ Couns.* 1999;38(1):33-42.
89. Teixeira PA, Gordon P, Camhi E, Bakken S. HIV patients' willingness to share personal health information electronically. *Patient Educ Couns.* 2011;84(2):e9-12.
90. Donenberg GR, Emerson E, Bryant FB, Wilson H, Weber-Shifrin E. Understanding AIDS-risk behavior among

adolescents in psychiatric care: links to psychopathology and peer relationships. *J Am Acad Child Adolesc Psychiatry.* 2001;40(6):642-653.
91. Garofalo R, Mustanski BS, McKirnan DJ, Herrick A, Donenberg GR. Methamphetamine and young men who have sex with men: understanding patterns and correlates of use and the association with HIV-related sexual risk. *Arch Pediatr Adolesc Med.* 2007;161(6):591-596.
92. Garofalo R, Herrick A, Mustanski BS, Donenberg GR. Tip of the Iceberg: young men who have sex with men, the Internet, and HIV risk. *Am J Public Health.* 2007;97(6):1113-1117.
93. Parsons JT, Halkitis PN, Bimbi D, Borkowski T. Perceptions of the benefits and costs associated with condom use and unprotected sex among late adolescent college students. *J Adolesc.* 2000;23(4):377-391.
94. Crosby RA, Sanders SA, Yarber WL, Graham CA, Dodge B. Condom use errors and problems among college men. *Sex Transm Dis.* 2002;29(9):552-557.
95. Tarnowski KJ, Simonian SJ. Assessing treatment acceptance: the Abbreviated Acceptability Rating Profile. *J Behav Ther Exp Psychiatry.* 1992;23(2):101-106.
96. Iivari J. An empirical test of the DeLone-McLean model of information system success. *SIGMIS Database.* 2005;36(2):8–27.
97. Bandura A. *Social cognitive theory and exercise control of HIV infection.* New York, NY: Plenum Press; 1994.
98. Meyer IH, Dean L. Patterns of sexual behavior and risk taking among young New York City gay men. *AIDS Educ Prev.* 1995;7(5 Suppl):13-23.
99. Carey MP, Schroder KE. Development and psychometric evaluation of the brief HIV Knowledge Questionnaire. *AIDS Educ Prev.* 2002;14(2):172-182.
100. National Institute on Drug Abuse. NIDA-modified ASSIST. In:2015.
101. Hennessy M, Bleakley A, Fishbein M, Jordan A. Validating an index of adolescent sexual behavior using psychosocial theory and social trait correlates. *AIDS Behav.* 2008;12(2):321-331.
102. Mansergh G, Koblin BA, McKirnan DJ, et al. An intervention to reduce HIV risk behavior of substance-using men who have sex with men: a two-group randomized trial with a nonrandomized third group. *PLoS Med.* 2010;7(8):e1000329.
103. Little R, Rubin D. *Statistical analysis with missing data.* New York, NY: Wiley; 1987.
