# Supplementary material for: Efficacy of MyPEEPS Mobile, an HIV Prevention Intervention Using Mobile Technology, on Reducing Sexual Risk Among Same-Sex Attracted Adolescent Males: A Randomized Clinical Trial
Source: JAMA Netw Open. 2022 Sep 21;5(9):e2231853. doi: 10.1001/jamanetworkopen.2022.31853 (PMC9494195; doi:10.1001/jamanetworkopen.2022.31853)
Supplement: Supplement 2. — eTable 1. Open and Closed Enrollment Time-Points eTable 2. Stratified Analysis of Condomless Sex Acts for Black or African American Non-Hispanic Participants eFigure 1. MyPEEPS Enrollment by Region eFigure 2. Sample MyPEEPS Content and Activities [file jamanetwopen-e2231853-s002.pdf]

Supplemental Online Content

Schnall R, Kuhns LM, Pearson C, et al. Efficacy of MyPEEPS Mobile, an HIV prevention intervention using mobile technology, on reducing sexual risk among same-sex attracted adolescent males. *JAMA Netw Open.* 2022;5(9):e2231853. doi:10.1001/jamanetworkopen.2022.31853

eTable 1. Open and Closed Enrollment Time-Points

eTable 2. Stratified Analysis of Condomless Sex Acts for Black or African American Non-Hispanic Participants

eFigure 1. MyPEEPS Enrollment by Region

eFigure 2. Sample MyPEEPS Content and Activities

This supplemental material has been provided by the authors to give readers additional information about their work.

© 2022 Schnall R et al. *JAMA Network Open.*

eTable 1. Open and Closed Enrollment Time-Points		
Date	Closed Enrollment	Re-Opened Enrollment
April 2019	White (Non-Hispanic/Latino), Ages 13-18	
September 2019	Black/African American (Non-Hispanic/Latino), Ages 15-18	White (Non-Hispanic/Latino), Ages 13-14[1]
November 2019		• White (Hispanic/Latino), Ages 15-18 from Rural Areas<br>• Black/African American (Hispanic/Latino), Ages 15-18 from Rural Areas
March 2020	• White (Hispanic/Latino or Non-Hispanic/Latino), Age 14 from Urban Areas<br>• Black/African American (Hispanic/Latino or Non-Hispanic/Latino), Age 14 from Urban Areas<br>• Asian (Hispanic/Latino or Non-Hispanic/Latino), Age 14-18 from Urban Areas	

© 2022 Schnall R et al. *JAMA Network Open.*

eTable 2. Stratified Analysis of Condomless Sex Acts for Black or African American Non-Hispanic Participants

	Condomless sex acts IRR (95% CI)
Time	
Baseline (Reference)	-
3-Months	2.72 (0.89, 8.31)
6-Months	2.62 (0.83, 8.31)
9-Months	4.57 (1.48, 14.15)
Intervention	2.35 (0.67, 8.22)
Intervention * Time	
Intervention * 3-Months	0.19 (0.04, 0.94)
Intervention * 6-Months	0.15 (0.03, 0.78)
Intervention * 9-Months	0.27 (0.06, 1.32)

Note. All models control for age, study site, and recruitment method (i.e., online / in person)

© 2022 Schnall R et al. *JAMA Network Open.*

eFigure 1. MyPEEPS Enrollment by Region

Western (Seattle)	Midwest (Chicago)	South (Birmingham)	Northeast (NYC)
1. California (CA)	1. Kansas (KS)	1. Alabama (AL)	1. Pennsylvania (PA)
2. Arizona (AZ)	2. Nebraska (NE)	2. Arkansas (AR)	2. New Jersey (NJ)
3. New Mexico (NM)	3. South Dakota (SD)	3. Delaware (DE)	3. New York (NY)
4. Nevada (NV)	4. North Dakota (ND)	4. Washington D.C. (DC)	4. Connecticut (CT)
5. Utah (UT)	5. Minnesota (MN)	5. Florida (FL)	5. Rhode Island (RI)
6. Colorado (CO)	6. Iowa (IA)	6. Georgia (GA)	6. Massachusetts (MA)
7. Oregon (OR)	7. Missouri (MO)	7. Kentucky (KY)	7. New Hampshire (NH)
8. Idaho (ID)	8. Illinois (IL)	8. Louisiana (LA)	8. Vermont (VT)
9. Wyoming (WY)	9. Wisconsin (WI)	9. Maryland (MD)	9. Maine (ME)
10. Washington (WA)	10. Indiana (IN)	10. Mississippi (MS)	
11. Montana (MT)	11. Michigan (MI)	11. North Carolina (NC)	
12. Hawaii (HI)	12. Ohio (OH)	12. Oklahoma (OK)	
13. Alaska (AK)		13. South Carolina (SC)	
		14. Tennessee (TN)	
		15. Texas (TX)	
		16. Virginia (VA)	
		17. West Virginia (WV)	

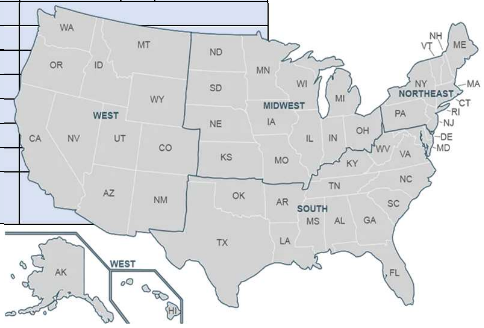

© 2022 Schnall R et al. *JAMA Network Open.*

eFigure 2. Sample MyPEEPS Content and Activities

User sets up their MyPEEPS profile and chooses their notification settings.	User completes a series of True/False questions related to HIV, with detailed fact-based information provided for each response.	User watches a video animation about what to expect during Tommy’s first experience being tested for HIV. Video presents clinic scenario and discussion with the HIV test counselor.
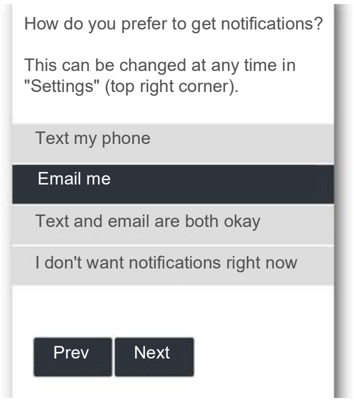	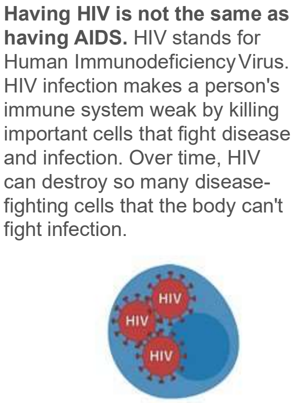	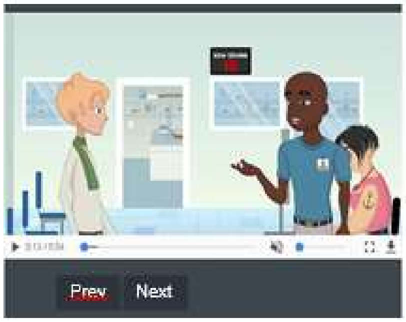
