# Supplementary material for: Efficacy of MyPEEPS Mobile, an HIV Prevention Intervention Using Mobile Technology, on Reducing Sexual Risk Among Same-Sex Attracted Adolescent Males: A Randomized Clinical Trial
Source: JAMA Netw Open. 2022 Sep 21;5(9):e2231853. doi: 10.1001/jamanetworkopen.2022.31853 (PMC9494195; doi:10.1001/jamanetworkopen.2022.31853)
Supplement: Supplement 3. — Data Sharing Statement [file jamanetwopen-e2231853-s003.pdf]

Data Sharing Statement

Schnall. Efficacy of MyPEEPS Mobile, an HIV Prevention Intervention Using Mobile Technology, on Reducing Sexual Risk Among Same-Sex Attracted Adolescent Males. *JAMA Netw Open*. Published September 21, 2022. doi:10.1001/jamanetworkopen.2022.31853

Data
Data available: No

Additional Information
Explanation for why data not available: Due to the sensitive information collected about our participants and their young age and the potential for being able to identify their identity, we would prefer not to make this data set publicly available but we are open to reviewing this decision with the editor.
